# Monsoon-driven assembly of the world’s largest evergreen broadleaved forest

**DOI:** 10.1093/nsr/nwag361

**Published:** 2026-06-12

**Authors:** Huan-Wen Peng, Lisi Hai, Xiao-Qian Li, Rosa del C Ortiz, Florian Jabbour, Wei Wang

**Affiliations:** State Key Laboratory of Plant Diversity and Specialty Crops, Institute of Botany, Chinese Academy of Sciences, Beijing 100093, China; China National Botanical Garden, Beijing 100093, China; State Key Laboratory of Plant Diversity and Specialty Crops, Institute of Botany, Chinese Academy of Sciences, Beijing 100093, China; School of Pharmacy, Jiangxi University of Chinese Medicine, Nanchang 330004, China; State Key Laboratory of Plant Diversity and Specialty Crops, Institute of Botany, Chinese Academy of Sciences, Beijing 100093, China; China National Botanical Garden, Beijing 100093, China; University of Chinese Academy of Sciences, Beijing 100049, China; Missouri Botanical Garden, St. Louis, MO 63110, USA; Institut de Systématique, Evolution, Biodiversité (ISYEB), Muséum national d’Histoire naturelle, CNRS, Sorbonne Université, Université des Antilles, EPHE, Paris 75005, France; State Key Laboratory of Plant Diversity and Specialty Crops, Institute of Botany, Chinese Academy of Sciences, Beijing 100093, China; China National Botanical Garden, Beijing 100093, China; University of Chinese Academy of Sciences, Beijing 100049, China

**Keywords:** biogeography, biome, biodiversity, East Asia, monsoon system

## Abstract

As the world’s largest evergreen broadleaved forest (EBLF), East Asian EBLF houses high levels of biodiversity and endemism and is essential to regional carbon storage and cycling. However, how East Asian EBLF was shaped over time remains an open question. Here, we investigate the historical assembly of this biome by sampling 21 angiosperm clades (together encompassing 2028 species) that include evergreen and deciduous species. We show that the transition from deciduous to evergreen lineages, and *in situ* diversification and immigration of evergreen lineages in subtropical East Asia all experienced a dynamic process. Transition and immigration reached their first peaks and *in situ* diversification sharply increased at the Oligocene–Miocene boundary (OMB), and all of them reached their highest peaks in the late Miocene. Our results suggest that modern East Asian EBLF did not appear until the OMB and markedly deteriorated since the Pliocene, mainly driven by the evolution of Asian monsoon climate. This study suggests that East Asian EBLF has functioned as both museums and cradles for regional biodiversity, and serves as a transfer station for biotic exchanges between temperate and tropical regions, highlighting its great conservation value.

## INTRODUCTION

**Figure 1.**
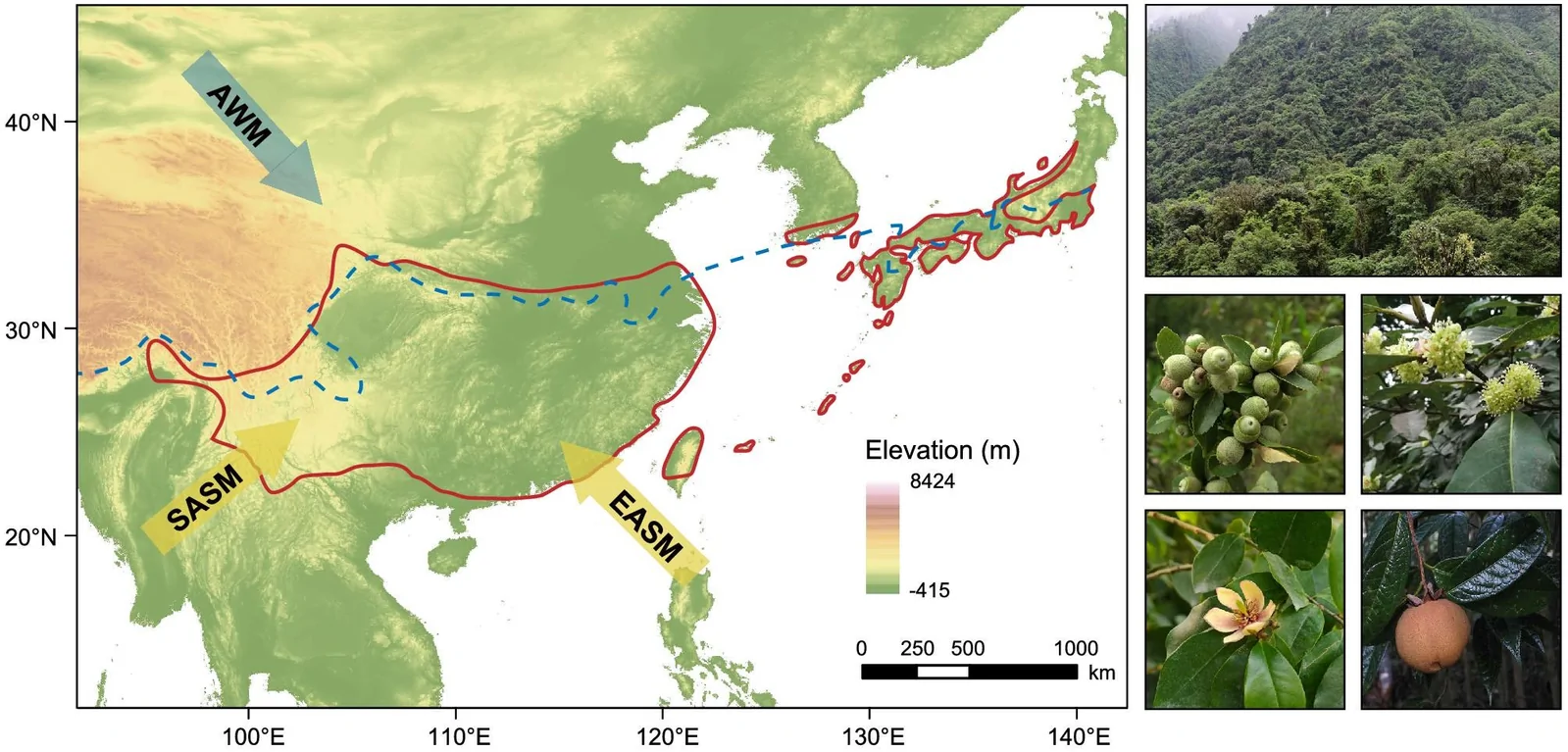
Geographic map of East Asian EBLF. The range of East Asian EBLF (solid line) is modified from Song and Da [5] and Tang [6]. The freezing boundary (dashed line) is here defined as having a mean minimum annual temperature ≤0°C (calculated from WorldClim data https://worldclim.org/). Representative landscape and plants are shown on the right: upper, EBLF in southeastern QTP; middle left, *Quercus cocciferoides* (Fagaceae); middle right, *Lindera megaphylla* (Lauraceae); bottom left, *Magnolia figo* (Magnoliaceae); bottom right, *Camellia crapnelliana* (Theaceae). AWM, Asian winter monsoon; EASM, East Asian summer monsoon; SASM, South Asian summer monsoon. Review drawing number: GS 京(2026)2003号.

Biodiversity is very unevenly distributed on Earth [1]. Understanding how biodiversity in an ecoregion or biome, especially species-rich, has developed over time is a fundamental question in ecology and evolutionary biology [2,3]. As the world’s largest evergreen broadleaved forest (EBLF) [4], East Asian EBLF covers southern China, southern Japan and southernmost Korea (Fig. 1) [5], and is characterized by high levels of species diversity and endemism compared to the regions at similar latitudes elsewhere on the planet, which are mostly arid and semiarid lands [6]. This biome is home to ∼2600 genera and 14 600 species of seed plants, of which >50% are endemics [7], with many relict species [8,9]. East Asian EBLF plays important roles in regional carbon storage and cycling, providing various biological resources, and promoting sustainable social development [10,11]. Nevertheless, the primeval EBLF in East Asia has been greatly diminished owing to anthropogenic activities [6,12] and only covers ∼5% of China’s total land area nowadays [13], underlining the urgency of understanding its historical assembly.

Unfortunately, the evolutionary history of East Asian EBLF remains contentious. Plant fossils and paleovegetational reconstructions suggest that this biome had emerged by the middle Eocene [14–16] or late Eocene–early Oligocene [17,18]. By integrating fossil data and modelling results, Li *et al.* [19] show that the transition from deciduous to evergreen habits in East Asian broadleaved forests occurred at the Oligocene–Miocene boundary (OMB). Dated phylogenies of representative components of East Asian EBLF support the rise of this biome in the Eocene [20–23], early Oligocene [24,25] or around the OMB [26–29]. Nevertheless, an organismal group could require a long timescale to adapt to a novel climatic regime or habitat, that is, an ‘evolutionary lag time’ often occurs [30]. This makes it challenging to draw a comprehensive picture of the historical assembly of a biome using a single taxon as the proxy. By analyzing the crown group ages of 72 so-called characteristic genera, Zhang *et al.* [31] suggest an early Oligocene origin for Chinese EBLF. These genera were selected based on the proportion of species in Chinese EBLF to the total number of species in China (≥20%) [31]. Under this criterion, monotypic or oligotypic genera were easily included, such as *Cercidiphyllum, Cyclocarya, Euptelea* and *Sargentodoxa*, but they are deciduous; moreover, some genera with many non-East Asian and/or non-evergreen species were also included, such as *Acer, Ilex* and *Viburnum*, but the original ages of the evergreen lineages in these genera are obviously younger than their crown group ages. ‘Evergreen’ is the most prominent feature for East Asian EBLF [5], and only the evolutionary dynamics of evergreen lineages can provide insights into the formation process of this biome [19,25–28,32]. To elucidate how East Asian EBLF was shaped over time, a molecular phylogenetic study of multiple clades with evergreen lineages across the angiosperm tree of life is essential.

Biome assembly is closely associated with geoclimatic changes [33,34]. The India–Eurasia collision started in the Paleocene and subsequently caused the deformation and uplift of the Qinghai–Tibet Plateau (QTP) [35,36], which have had a profound impact on regional and even global climates and environments [37,38]. The data from ^δ18^O levels in benthic foraminifera show that global temperature changed dramatically in the Cenozoic [39]. Importantly, the Asian monsoon climate, which can bring abundant rainfall, is generally considered to be the most important factor that influences the evolutionary process of East Asian EBLF [26–28]. Yet, the evolution of the Asian monsoon system was complex during the Cenozoic [37,40]. Some studies suggest that the Asian monsoon climate resembling the modern pattern was fully established around the OMB [38,41]. In contrast, other studies support that the Asian monsoon had emerged by the Eocene [42,43] or even Paleocene [44]. Nevertheless, tropical monsoon reached only low latitude regions in Asia (south of 20–22°N) due to seasonal fluctuation of the Intertropical Convergence Zone (ITCZ) in the early Cenozoic, and until ∼41 Ma, the Asian monsoon did not expand northward to subtropical southern China [38,45].

Here, we attempt to connect past geoclimatic changes to the biological processes that have driven the assembly of East Asian EBLF. We use evergreen lineages within the 21 selected angiosperm clades as the proxy to unravel the evolutionary history of East Asian EBLF at a global scale, by conducting a multi-taxon analysis. We investigate whether phylogenetic estimates of East Asian evergreen ancestry are temporally in agreement with the formation of modern-like Asian monsoon climate, and test whether geoclimatic events in East Asia left discernible imprints in the assembly of East Asian EBLF. Specially, we test whether the transition events from deciduous to evergreen habits in subtropical East Asia mainly took place around the OMB or experienced a dynamic process. We further investigate the temporal dynamics of *in situ* diversification and immigration events of evergreen lineages in East Asian EBLF. We show that modern-like East Asian EBLF began to appear around the OMB, flourished continuously throughout the Miocene and deteriorated since the Pliocene, which were mainly influenced by a long-term and complex evolution of Asian monsoon system. We further discover that East Asian EBLF is both evolutionary cradles and museums for East Asian biodiversity and serves as a transfer station for biotic exchanges between temperate and tropical regions.

## RESULTS AND DISCUSSION

To illustrate the assembly process of East Asian EBLF, we selected 21 clades with 2028 species, belonging to 14 orders and 24 families across the angiosperm tree of life (Fig. S1 and Table S1). A total of 680 evergreen species (406 endemic) and 381 deciduous species (206 endemic) growing in subtropical East Asia are included. This sampling strategy maximized taxon representation in East Asian EBLF so far, especially covering the members of the first five dominant families in this biome, i.e. Fagaceae, Lauraceae, Magnoliaceae, Theaceae and Hamamelidaceae [5]. By integrating results of molecular dating, biogeographic and habit analyses for each of the 21 clades (Fig. S2), we compiled age credibility intervals of the transition from deciduous to evergreen habits in subtropical East Asia, and calculated the maximal number of observed transition events (MTE) per million year (Myr) (Fig. S3). Considering that the species diversity in a biome arises from both *in situ* diversification and immigration, we further compiled credibility intervals of age estimates for *in situ* diversification and dispersal events of evergreen lineages and calculated their maximal number of observed events (MDivE & MDisE) per Myr, respectively. In addition, we performed generalized least squares multiple regression analyses to test the possible correlation between evolutionary dynamics of evergreen lineages and environmental changes, i.e. East Asian annual precipitation and global temperature, over time and employed breakpoint regression analyses to test whether Asian monsoon climate change impacted the MTE, MDivE and MDisE.

**Figure 2.**
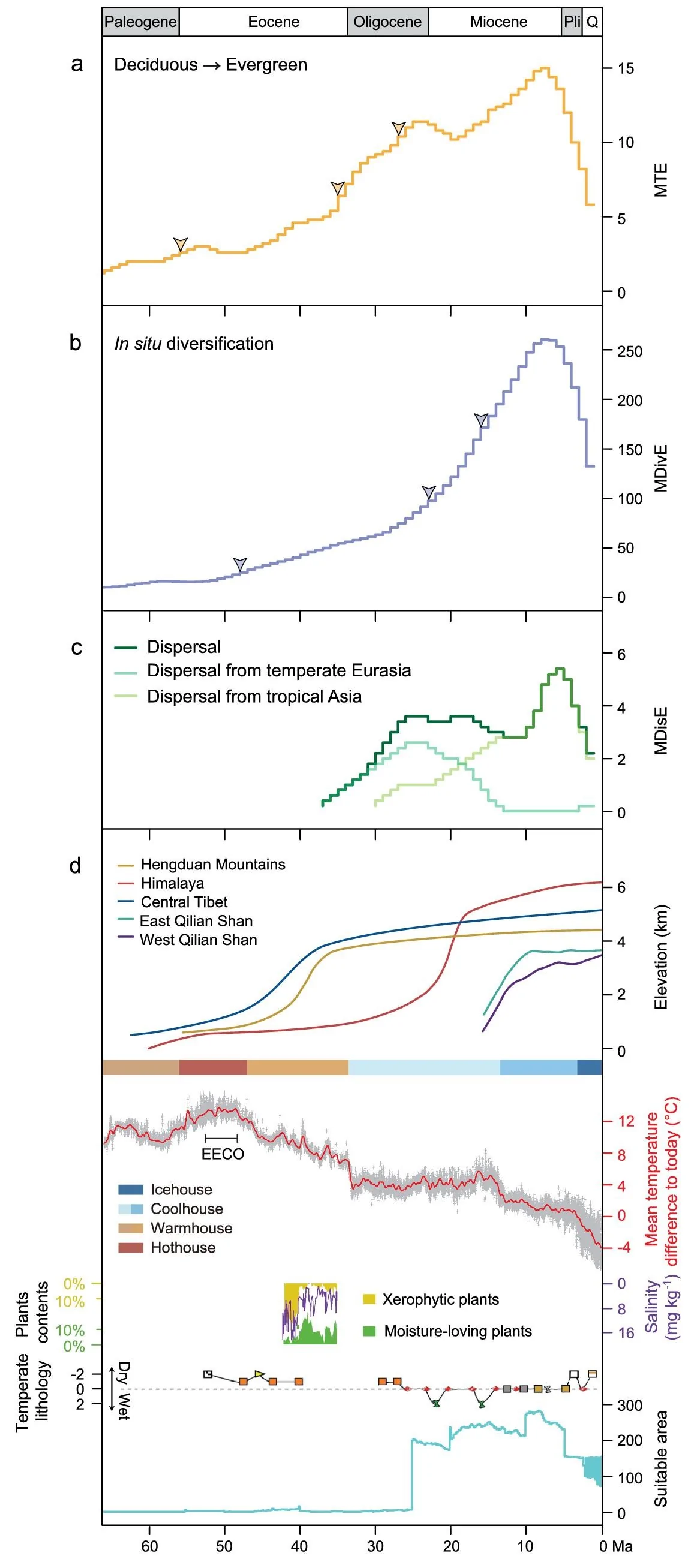
Evolutionary dynamics of evergreen lineages in subtropical East Asia and potential driving factors. (a) Transition rates from deciduous to evergreen habits based on the MTE per Myr. (b) *In situ* diversification rates of evergreen lineages based on the MDivE per Myr. Details for transition and *in situ* diversification dynamics in a broader timeframe can be found in Fig. S3. Arrowheads indicate estimated inflection points. (c) Dispersal rates of evergreen lineages from other regions to subtropical East Asia based on the MDisE per Myr. (d) Elevation changes of different parts of the QTP [35] and the Qilian Shan [56], global mean temperature difference to today [39], Asian climatic records [38] and suitable area (grid cell) dynamics of East Asian EBLF. EECO, Early Eocene Climatic Optimum; Pli, Pliocene; Q, Quaternary.

**Figure 3.**
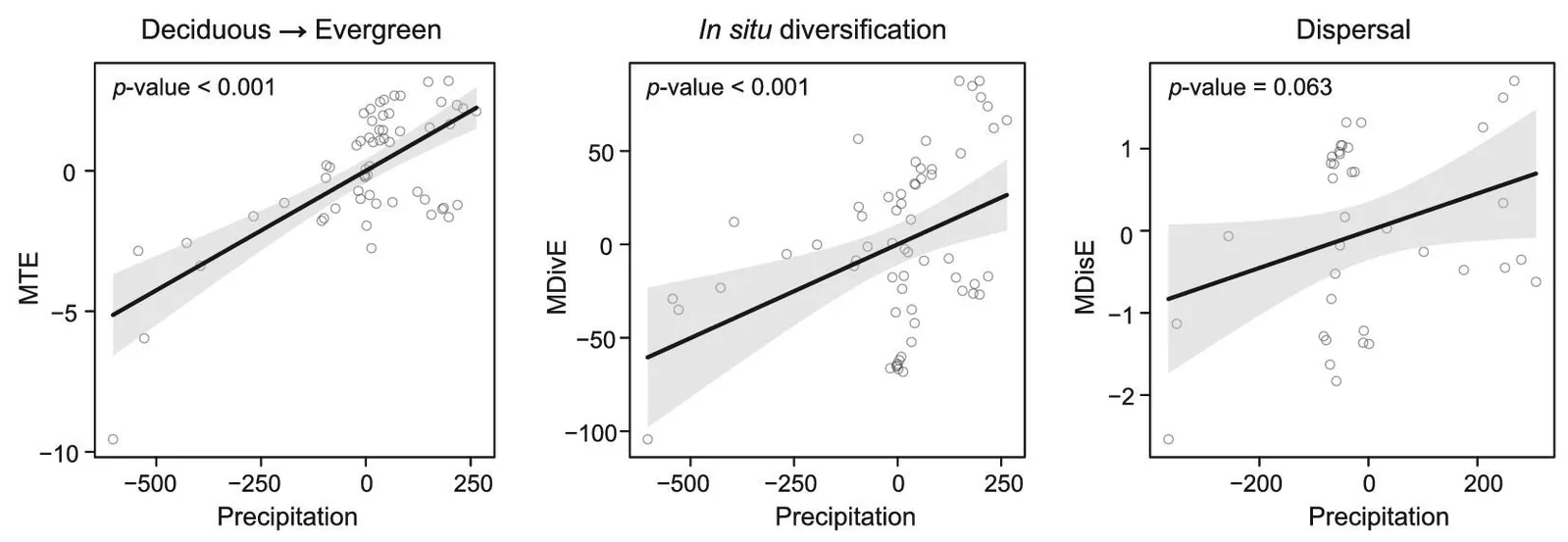
Partial regression plots showing correlations between the three events related to East Asian subtropical evergreen lineages and annual precipitation in East Asia. The solid line and gray shading indicate the regression line and 95% credible interval separately, with circles showing the raw data.

Our biogeographic and habit analyses identified 48 transition events from deciduous to evergreen habits in subtropical East Asia. A total of 788 biogeographic events related to subtropical East Asian evergreen species examined here were found, including 773 *in situ* diversification events and 15 dispersal events. *In situ* diversification events overwhelmingly predominated over dispersal events by ∼52 times (773/15), implying that indigenous elements contributed far more than immigrants to the current biodiversity of East Asian EBLF. Considering the relatively low sampling for some clades, this hypothesis needs to be tested in the future by sampling more taxa. The transition from deciduous to evergreen habits and *in situ* diversification initially occurred in the Lower Cretaceous (Fig. S3). Transition events accelerated at ∼56, 35 and 27 Ma, and peaked around 25–23 Ma and 8–7 Ma (Fig. 2a). *In situ* diversification events accelerated at ∼48, 23 and 16 Ma, and peaked around 8–6 Ma (Fig. 2b). Dispersal events into subtropical East Asia started at ∼36 Ma and peaked around 26–23 Ma and 6–5 Ma, which were from two source regions: temperate Eurasia and tropical Asia (Fig. 2c). Our multiple regression analyses support the positive relationship between evergreen lineage occurrence, *in situ* diversification, dispersal and East Asian annual precipitation, respectively (Fig. 3 and Table S2), highlighting the important roles of the precipitation regime in promoting the diversification of evergreen lineages.

*In situ* diversification began at ∼125 Ma, by ancestral lineage of the Altingiaceae–Hamamelidaceae clade (Figs S2 and S3). Transition started at ∼93 Ma, mainly by ancestral lineage of *Magnolia* (Figs S2 and S3 and Table S3). These suggest that some ancient elements of East Asian EBLF might have occurred in the Lower to Upper Cretaceous, implying that the EBLF functions as a museum for plant species diversity in East Asia. During this period, global average temperature was considerably high without large and permanent icecaps [46], and angiosperm-dominated forests started to arise [47,48] along with the Cretaceous Terrestrial Revolution [49].

The MTE curve initially accelerated at ∼56 Ma (Fig. 2a), which temporally coincides with the transition from the ‘Warmhouse’ to ‘Hothouse’ climate state (Fig. 2d) [39]. The MDivE curve first accelerated at ∼48 Ma (Fig. 2b), in line with the Early Eocene Climatic Optimum (Fig. 2d). Geological data indicate the uplift of southern (∼55 Ma) and central (∼45 Ma) parts of the QTP caused by the India–Eurasia collision [35]. The orogenic events in the QTP, as well as the migration of Pacific warm pool back to Asia, resulted in the dominance of ITCZ-type monsoonal climate in East Asia and thereby brought more precipitation [37]. We also identified an increased point of the MTE curve in the late Eocene (∼35 Ma; Fig. 2a). During this period, subtropical monsoon climate gradually prevailed in southern China and expanded northward (Fig. 2d), along with significant increase in precipitation [38,45,50,51]. Based on our ancestral range and habit reconstructions (Fig. S2), most evergreen lineages that originated in the Eocene are currently distributed in southern China to Southeast Asia, such as some species of *Lithocarpus* (Fagaceae), *Actinodaphne henryi* and *Sinosassafras flavinervium* (Lauraceae) and the *Viburnum punctatum*–*V. lepidotulum* clade (Viburnaceae). Thus, EBLF might have only appeared in low latitudes of subtropical East Asia during the Eocene. Paleobotanical data also indicate that dominant genera in southern Chinese EBLF started to occur in the Paleocene–early Eocene, and peaked in the middle Eocene [16].

**Figure 4.**
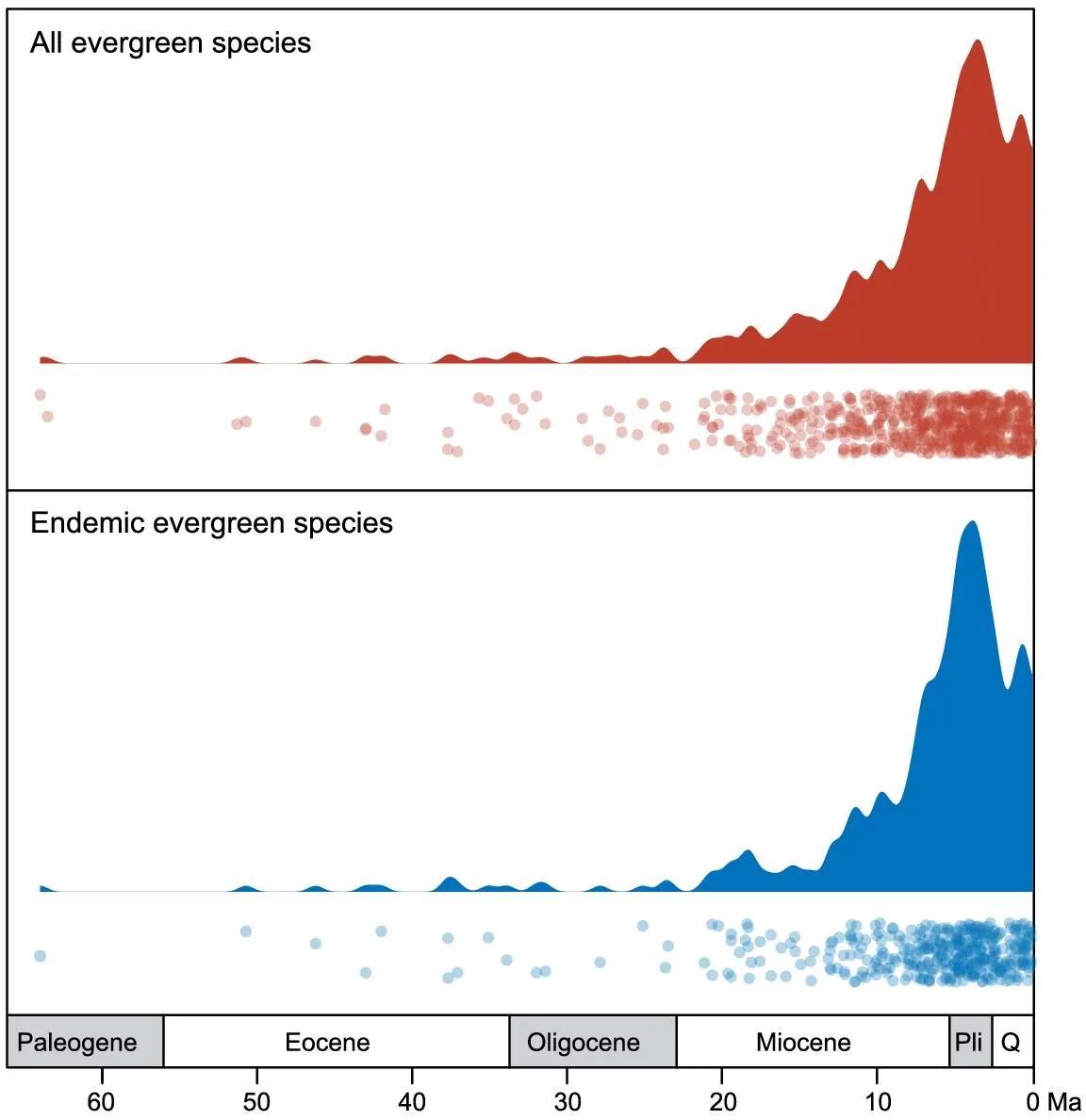
Distribution of estimated origination ages of evergreen species in East Asian EBLF.

Transition further accelerated in the late Oligocene (∼27 Ma) and later reached the first peak around 25–23 Ma (Fig. 2a). The MDivE curve sharply increased at ∼23 Ma (Fig. 2b), and the MDisE curve peaked around 26–23 Ma (Fig. 2c). Moreover, MDivE and MDisE curves that were calculated based on the evergreen lineages only from the first five dominant families in East Asian EBLF significantly increased around the OMB (Fig. S4). In the late Oligocene–early Miocene, the dramatic uplift of central and southern QTP occurred (Fig. 2d), which largely reorganized Asian climate and shaped the modern-like Asian monsoon system (Fig. 2d) [38,41]. During this period, East Asian summer monsoon started to establish [52], and South Asian summer monsoon intensified [53], contributing to more summer precipitation [54] and the emergence of spring persistent rainfall [40] in East Asia. Meanwhile, the weakening of Asian winter monsoon increased winter precipitation in East Asia [19]. The breakpoint regression analyses supported that the formation of modern-like Asian monsoon system influenced all of the MTE, MDivE and MDisE (Table 1). Our modelling analysis indicates significant increase of suitable area of East Asian EBLF at ∼25 Ma (Fig. 2d), in agreement with the result of Guo *et al.* [55] that a wide humid belt similar to the modern one occurred in subtropical East Asia near the OMB. Importantly, considering the credibility intervals of estimated times, 85%, 88% and 93% of transition, *in situ* diversification and dispersal events took place after the OMB (∼23 Ma). We also found that ∼95% of the evergreen species in East Asian EBLF and ∼96% of the evergreen species endemic to this biome occurred after ∼23 Ma (Fig. 4), suggesting that East Asia EBLF acted as an evolutionary cradle for East Asian biodiversity. Thus, our data show that the modified monsoon system resulted in the humidification of East Asia and thereby promoted the modernization of the EBLF in this region around the OMB, as supported by the results of modelling and fossil data [19].

**Table 1.** Model evaluation for breakpoint regression analyses using Bayesian inference on the temporal dynamics of transition (MTE), *in situ* diversification (MDivE) and dispersal (MDisE) events. Model description: Null, model with no break points; E, model with a break point around ∼41 Ma, representing northward expansion of Asian monsoon to subtropical southern China; M, model with a break point between 25 and 23 Ma, representing formation of modern-like Asian monsoon system; P, model with a break point between 3.6 and 2.6 Ma, representing decrease in East Asian precipitation. Models with an estimated log posterior density (ELPD) difference of zero are considered to be the optimal model (in bold).

	ELPD difference ± standard error		
Model	MTE	MDivE	MDisE
Null	−36.8 ± 6.6	−41.4 ± 5.8	−13.2 ± 4.6
E	−7.0 ± 8.8	−27.1 ± 5.4	N/A
**M**	−4.6 ± 3.2	**0.0 ± 0.0**	−0.2 ± 1.7
P	−37.8 ± 6.6	−40.9 ± 5.8	−14.0 ± 4.5
EM	−16.9 ± 7.8	−26.7 ± 5.4	N/A
EP	−5.8 ± 9.1	−27.8 ± 5.8	N/A
**MP**	**0.0 ± 0.0**	−0.2 ± 1.3	**0.0 ± 0.0**
EMP	−5.8 ± 9.0	−27.8 ± 5.8	N/A

The MDivE curve shows a virtually linear increase between 23 and 8 Ma (Fig. 2b) and peaked in the late Miocene (∼8–5 Ma), during which MTE and MDisE curves also reached their high peaks (Fig. 2a–c). This temporally coincides with the uplift of Qilian Shan (Fig. 2d) [56]. From ∼12 to 4 Ma, an intensified Asian monsoon dominated East Asia, which might have contributed to the more persistent wet season through the year [37,51], and the East Asian spring persistent rainfall reached its modern-day geographic distribution [40]. The EBLF thereby flourished continuously in East Asia throughout the Miocene.

From ∼4 Ma onwards, the MTE, MDivE and MDisE curves all decreased (Fig. 2a–c), which temporally coincides with a marked decrease of precipitation in East Asia [37,40] and the transition from the ‘Coolhouse’ to ‘Coldhouse’ climate state (Fig. 2d). The freezing temperature is a key factor limiting the distribution of evergreen broadleaved species [5,28]. The breakpoint regression analyses also found that this decrease in precipitation had an impact on the MTE and MDisE curves (Table 1). In addition, our modelling results suggest the significant decrease of suitable areas of East Asian EBLF occurred at this time (Fig. 2d). Thus, temperature and precipitation drop could have hampered the diversification of evergreen lineages and have progressively deteriorated East Asian EBLF since the Pliocene.

Among the referred 15 dispersal events of evergreen lineages into subtropical East Asia (Table S4), most of them were from tropical Asia (∼73%), and the remaining were from temperate Eurasia (∼27%), suggesting the great contribution of tropical Asia to species diversity of East Asian EBLF. In addition, we also identified 225 dispersal events out of East Asian EBLF spanning from 90.42 to 0.29 Ma, most of which (∼81.3%) colonized tropical Asia, followed by temperate Eurasia (∼14.7%), North America (∼2.2%), South America (∼1.3%) and Africa (∼0.4%) (Fig. S5). Altogether, these results suggest that East Asian EBLF serves as not only a sink, but also an important source region for species diversity of other regions of the world, and thus promotes biotic exchanges between temperate and tropical regions, highlighting the importance of its conservation.

In summary, our multi-taxon study provides new insights into the historical assembly of East Asian EBLF over a broad period of ∼125 Myr. All of the MTE, MDivE and MDisE curves experienced a dynamic process. The occurrence of *in situ* diversification is much earlier than that of dispersal, and the former overwhelmingly predominated over the latter. Our results support that the modernization of East Asian EBLF did not take place until the OMB, which was driven by the formation of modern-like Asian monsoon climate system. East Asian EBLF has functioned as both evolutionary museums and cradles for evergreen species diversity of East Asia, and served as a transfer station for biotic exchanges between temperate and tropical regions, emphasizing the conservation priority for this world’s largest EBLF. Many herbaceous and deciduous species also inhabit East Asian EBLF. Whether they have similar trends through time to evergreen species needs to be investigated in the future.

## MATERIALS AND METHODS

Detailed materials and methods are available in the Supplementary material.

## Supplementary Material

nwag361_Supplemental_File

## Data Availability

The data underlying this article are available at Figshare (https://doi.org/10.6084/m9.figshare.30162892).

## References

[1] Marchese C . Biodiversity hotspots: a shortcut for a more complicated concept. Glob Ecol Conserv 2015; 3: 297–309.

[2] Darwin C . The Origin of Species by Means of Natural Selection. London: Murray, 1859.

[3] Pennington RT, Richardson JE, Lavin M. Insights into the historical construction of species-rich biomes from dated plant phylogenies, neutral ecological theory and phylogenetic community structure. New Phytol 2006; 172: 605–16. 10.1111/j.1469-8137.2006.01902.x 17096788

[4] Song YC . Evergreen Broad-Leaved Forests in China: Classification-Ecology-Conservation. Beijing: Science Press, 2013.

[5] Song YC, Da LJ. Evergreen broad-leaved forest of East Asia. In: Box EO (ed.). Vegetation Structure and Function at Multiple Spatial, Temporal and Conceptual Scales. Cham: Springer, 2016, 101–28.

[6] Tang CQ . The Subtropical Vegetation of Southwestern China: Plant Distribution, Diversity and Ecology. Dordrecht: Springer, 2015.

[7] Wu ZY . Vegetation of China. Beijing: Science Press, 1980.

[8] Manchester SR, Chen ZD, Lu AM et al. Eastern Asian endemic seed plant genera and their paleogeographic history throughout the Northern Hemisphere. J Syst Evol 2009; 47: 1–42. 10.1111/j.1759-6831.2009.00001.x

[9] Tang CQ, Matsui T, Ohashi H et al. Identifying long-term stable refugia for relict plant species in East Asia. Nat Commun 2018; 9: 4488. 10.1038/s41467-018-06837-3 30367062 PMC6203703

[10] Pan Y, Birdsey RA, Fang J et al. A large and persistent carbon sink in the world’s forests. Science 2011; 333: 988–93. 10.1126/science.1201609 21764754

[11] Fang J, Guo Z, Hu H et al. Forest biomass carbon sinks in East Asia, with special reference to the relative contributions of forest expansion and forest growth. Glob Change Biol 2014; 20: 2019–30. 10.1111/gcb.12512 24464906

[12] Wang XH, Kent M, Fang XF. Evergreen broad-leaved forest in Eastern China: its ecology and conservation and the importance of resprouting in forest restoration. Forest Ecol Manag 2007; 245: 76–87. 10.1016/j.foreco.2007.03.043

[13] Chen WL . The present status of degraded ecosystems in China. In: Chen LZ, Chen WL (eds). Degraded Ecosystems in China. Beijing: China Science and Technology Press, 1995, 16–23.

[14] Spicer RA, Herman AB, Liao W et al. Cool tropics in the Middle Eocene: evidence from the Changchang Flora, Hainan Island, China. Palaeogeogr Palaeoclimatol Palaeoecol 2014; 412: 1–16. 10.1016/j.palaeo.2014.07.011

[15] Herman AB, Spicer RA, Aleksandrova GN et al. Eocene–early Oligocene climate and vegetation change in southern China: evidence from the Maoming Basin. Palaeogeogr Palaeoclimatol Palaeoecol 2017; 479: 126–37. 10.1016/j.palaeo.2017.04.023

[16] Zhao J, Li S, Huang J et al. Heterogeneous occurrence of evergreen broad-leaved forests in East Asia: evidence from plant fossils. Plant Divers 2025; 47: 1–12. 10.1016/j.pld.2024.07.004 40041559 PMC11873578

[17] Su T, Spicer RA, Li SH et al. Uplift, climate and biotic changes at the Eocene–Oligocene transition in south-eastern Tibet. Natl Sci Rev 2019; 6: 495–504. 10.1093/nsr/nwy062 34691898 PMC8291530

[18] Tian Y, Spicer RA, Huang J et al. New early Oligocene zircon U-Pb dates for the ‘Miocene’ Wenshan Basin, Yunnan, China: biodiversity and paleoenvironment. Earth Planet Sci Lett 2021; 565: 116929. 10.1016/j.epsl.2021.116929

[19] Li SF, Valdes PJ, Farnsworth A et al. Orographic evolution of northern Tibet shaped vegetation and plant diversity in eastern Asia. Sci Adv 2021; 7: eabc7741. 33571113 10.1126/sciadv.abc7741 PMC7840128

[20] Huang JF, Li L, van der Werff H et al. Origins and evolution of cinnamon and camphor: a phylogenetic and historical biogeographical analysis of the *Cinnamomum* group (Lauraceae). Mol Phylogenet Evol 2016; 96: 33–44. 10.1016/j.ympev.2015.12.007 26718058

[21] Rao M, Steinbauer MJ, Xiang X et al. Environmental and evolutionary drivers of diversity patterns in the tea family (Theaceae s.s.) across China. Ecol Evol 2018; 8: 11663–76. 10.1002/ece3.4619 30598765 PMC6303774

[22] Hipp AL, Manos PS, Hahn M et al. Genomic landscape of the global oak phylogeny. New Phytol 2020; 226: 1198–212. 10.1111/nph.16162 31609470

[23] Dong S, Wang Y, Xia N et al. Plastid and nuclear phylogenomic incongruences and biogeographic implications of *Magnolia* s.l. (Magnoliaceae). J Syt Evol 2022; 60: 1–15. 10.1111/jse.12727

[24] Xiang XG, Mi XC, Zhou HL et al. Biogeographical diversification of mainland Asian *Dendrobium* (Orchidaceae) and its implications for the historical dynamics of evergreen broad-leaved forests. J Biogeogr 2016; 43: 1310–23. 10.1111/jbi.12726

[25] Deng M, Jiang XL, Hipp AL et al. Phylogeny and biogeography of East Asian evergreen oaks (*Quercus* section *Cyclobalanopsis*; Fagaceae): insights into the Cenozoic history of evergreen broad-leaved forests in subtropical Asia. Mol Phylogenet Evol 2018; 119: 170–81. 10.1016/j.ympev.2017.11.003 29175095

[26] Yu XQ, Gao LM, Soltis DE et al. Insights into the historical assembly of East Asian subtropical evergreen broadleaved forests revealed by the temporal history of the tea family. New Phytol 2017; 215: 1235–48. 10.1111/nph.14683 28695680

[27] Chen XH, Xiang KL, Lian L et al. Biogeographic diversification of *Mahonia* (Berberidaceae): implications for the origin and evolution of East Asian subtropical evergreen broadleaved forests. Mol Phylogenet Evol 2020; 151: 106910. 10.1016/j.ympev.2020.106910 32702526

[28] Hai L, Li XQ, Zhang J et al. Assembly dynamics of East Asian subtropical evergreen broadleaved forests: new insights from the dominant Fagaceae trees. J Integr Plant Biol 2022; 64: 2126–34. 10.1111/jipb.13361 36083596

[29] Xiao TW, Yan HF, Ge XJ. Plastid phylogenomics of tribe Perseeae (Lauraceae) yields insights into the evolution of East Asian subtropical evergreen broad-leaved forests. BMC Plant Biol 2022; 22: 32. 10.1186/s12870-021-03413-8 35027008 PMC8756638

[30] Guerrero PC, Rosas M, Arroyo MTK et al. Evolutionary lag times and recent origin of the biota of an ancient desert (Atacama–Sechura). Proc Natl Acad Sci USA 2013; 110: 11469–74. 10.1073/pnas.1308721110 23798420 PMC3710863

[31] Zhang Q, Yang Y, Liu B et al. Meta-analysis provides insights into the origin and evolution of East Asian evergreen broad-leaved forests. New Phytol 2024; 242: 2369–79. 10.1111/nph.19524 38186378

[32] Qin SY, Zuo ZY, Guo C et al. Phylogenomic insights into the origin and evolutionary history of evergreen broadleaved forests in East Asia under Cenozoic climate change. Mol Ecol 2023; 32: 2850–68. 10.1111/mec.16904 36847615

[33] Ding WN, Ree RH, Spicer RA et al. Ancient orogenic and monsoon-driven assembly of the world’s richest temperate alpine flora. Science 2020; 369: 578–81. 10.1126/science.abb4484 32732426

[34] Zhang J, Li XQ, Peng HW et al. Evolutionary history of the Arctic flora. Nat Commun 2023; 14: 4021. 10.1038/s41467-023-39555-6 37463899 PMC10354081

[35] Ding L, Kapp P, Cai F et al. Timing and mechanisms of Tibetan Plateau uplift. Nat Rev Earth Environ 2022; 3: 652–67. 10.1038/s43017-022-00318-4

[36] Cao GL, Li XQ, Xiang KL et al. Formation of three great Asian plateaus, climate change, and biodiversity. Trends Ecol Evol 2025; 40: 970–82. 10.1016/j.tree.2025.07.008 40803961

[37] Farnsworth A, Lunt DJ, Robinson SA et al. Past East Asian monsoon evolution controlled by paleogeography, not CO_2_. Sci Adv 2019; 5: eaax1697. 10.1126/sciadv.aax1697 31692956 PMC6821471

[38] Wu F, Fang X, Yang Y et al. Reorganization of Asian climate in relation to Tibetan Plateau uplift. Nat Rev Earth Environ 2022; 3: 684–700. 10.1038/s43017-022-00331-7

[39] Westerhold T, Marwan N, Drury AJ et al. An astronomically dated record of Earth’s climate and its predictability over the last 66 million years. Science 2020; 369: 1383–7. 10.1126/science.aba6853 32913105

[40] He L, Zhou T, Guo Z et al. Cenozoic evolution of spring persistent rainfall in East Asia and North America driven by paleogeography. Commun Earth Environ 2025; 6: 155. 10.1038/s43247-025-02136-0

[41] Spicer RA, Farnsworth A, Su T et al. The progressive co-evolutionary development of the Pan-Tibetan Highlands, the Asian monsoon system and Asian biodiversity. Geol Soc Spec Publ 2025; 549: SP549–2023–180. 10.1144/SP549-2023-180

[42] Spicer RA, Yang J, Herman AB et al. Asian Eocene monsoons as revealed by leaf architectural signatures. Earth Planet Sci Lett 2016; 449: 61–8. 10.1016/j.epsl.2016.05.036

[43] Xiong Z, Ding L, Spicer RA et al. The early Eocene rise of the Gonjo Basin, SE Tibet: from low desert to high forest. Earth Planet Sci Lett 2020; 543: 116312. 10.1016/j.epsl.2020.116312

[44] Ding L, Xu Q, Yue Y et al. The Andean-type Gangdese Mountains: paleoelevation record from the Paleocene–Eocene Linzhou Basin. Earth Planet Sci Lett 2014; 392: 250–64. 10.1016/j.epsl.2014.01.045

[45] Xie Y, Wu F, Miao Y et al. Reappraisal of Eocene climate patterns in East Asia: a synthetic review. Earth Sci Rev 2025; 271: 105281. 10.1016/j.earscirev.2025.105281

[46] Scotese CR, Song H, Mills BJW et al. Phanerozoic paleotemperatures: the earth’s changing climate during the last 540 million years. Earth-Sci Rev 2021; 215: 103503. 10.1016/j.earscirev.2021.103503

[47] Wang H, Moore MJ, Soltis PS et al. Rosid radiation and the rapid rise of angiosperm-dominated forests. Proc Natl Acad Sci USA 2009; 106: 3853–8. 10.1073/pnas.0813376106 19223592 PMC2644257

[48] Peng HW, Xiang KL, Erst AS et al. A complete genus-level phylogeny reveals the Cretaceous biogeographic diversification of the poppy family. Mol Phylogenet Evol 2023; 181: 107712. 10.1016/j.ympev.2023.107712 36693534

[49] Lloyd GT, Davis KE, Pisani D et al. Dinosaurs and the Cretaceous Terrestrial Revolution. Proc R Soc B 2008; 275: 2483–90. 10.1098/rspb.2008.0715 PMC2603200 18647715

[50] Zheng H, Yang Q, Cao S et al. From desert to monsoon: irreversible climatic transition at ∼ 36 Ma in southeastern Tibetan Plateau. Prog Earth Planet Sci 2022; 9: 12. 10.1186/s40645-022-00470-x

[51] Wu F, Tang F, Gao S et al. Northward expansion of Cenozoic Asian humid climate recorded by sporopollen. Palaeogeogr Palaeoclimatol Palaeoecol 2024; 637: 112009. 10.1016/j.palaeo.2023.112009

[52] Ding W, Hou D, Gan J et al. Palaeovegetation variation in response to the late Oligocene-early Miocene East Asian summer monsoon in the Ying-Qiong Basin, South China Sea. Palaeogeogr Palaeoclimatol Palaeoecol 2021; 567: 110205. 10.1016/j.palaeo.2020.110205

[53] Clift PD, Webb AAG. A history of the Asian monsoon and its interactions with solid Earth tectonics in Cenozoic South Asia. In: Treloar PJ, Searle M (eds). Himalayan Tectonics: A Modern Synthesis. London: Geological Society, 2019, 631–52.

[54] An Z, Sun Y, Chang H et al. Late Cenozoic climate change in monsoon-arid Asia and global changes. In: An Z (ed.). Late Cenozoic Climate Change in Asia: Loess, Monsoon and Monsoon-arid Environment Evolution. Dordrecht: Springer Netherlands, 2014, 491–581. 10.1007/978-94-007-7817-7_6

[55] Guo ZT, Sun B, Zhang ZS et al. A major reorganization of Asian climate by the early Miocene. Clim Past 2008; 4: 153–74. 10.5194/cp-4-153-2008

[56] Miao Y, Fang X, Sun J et al. A new biologic paleoaltimetry indicating Late Miocene rapid uplift of northern Tibet Plateau. Science 2022; 378: 1074–9. 10.1126/science.abo2475 36480632

