# Supplementary material for: Monsoon-driven assembly of the world’s largest evergreen broadleaved forest
Source: Natl Sci Rev. 2026 Jun 12;13(16):nwag361. doi: 10.1093/nsr/nwag361 (PMC13499221; doi:10.1093/nsr/nwag361)
Supplement: Supplementary file 1 [file nwag361_supplemental_file.doc]

**Supplementary Information for**

**Monsoon-driven assembly of the world’s largest evergreen broadleaved forest**

Huan-Wen Peng^1,2,†^, Lisi Hai^1,3,†^, Xiao-Qian Li^1,2,4^, Rosa del C. Ortiz^5^, Florian Jabbour^6^, Wei Wang^1,2,4,*^

^1^State Key Laboratory of Plant Diversity and Specialty Crops, Institute of Botany, Chinese Academy of Sciences, Beijing 100093, China; ^2^China National Botanical Garden, Beijing 100093, China; ^3^School of Pharmacy, Jiangxi University of Chinese Medicine, Nanchang 330004, China; ^4^University of Chinese Academy of Sciences, Beijing 100049, China; ^5^Missouri Botanical Garden, 4344 Shaw Boulevard, St. Louis, MO 63110, USA; ^6^Institut de Systématique, Evolution, Biodiversité (ISYEB), Muséum national d’Histoire naturelle, CNRS, Sorbonne Université, Université des Antilles, EPHE, 57 rue Cuvier, CP39, Paris 75005, France.

***Corresponding author.** E-mail: [wangwei1127@ibcas.ac.cn](mailto:wangwei1127@ibcas.ac.cn)

^†^Equally contributed to this work.

**This PDF file includes:**

Materials and Methods

Figures S1 to S6

Tables S1 to S6

References

**MATERIALS AND METHODS**

**Data collection**

We selected 21 angiosperm clades that contain both evergreen and deciduous species, are distributed in subtropical East Asia and adjacent regions, and have sufficient molecular data available to infer a time-calibrated phylogeny covering the major taxonomic and geographical diversity of the clade (Table S1). The 21 clades include 2028 sampled species, of which 680 are evergreen species that are distributed in East Asian evergreen broadleaved forest and 406 are endemic to this biome. These selected taxa comprise species with a wide variety of phylogenetic backgrounds, belonging to 14 orders and 24 families of angiosperms (Fig. S1, Table S1). For seven clades, we used the published chronograms from our previous studies (Fagaceae [1], Lardizabalaceae [2], and Magnoliaceae [3]) or other studies (Aquifoliaceae [4], *Celtis* [5], *Rhododendron* [6], and *Viburnum* [7]). For the remaining 14 clades, we conducted new phylogenetic and molecular dating analyses using published DNA datasets or sequence data downloaded from GenBank (Table S5). The sequences of each locus were aligned using MAFFT v.7 [8] with manual adjustment in Geneious v.8.0.4 [9], in which poorly aligned regions were removed automatically with trimAl [10].

**Phylogenetic analysis and molecular dating**

For each of the 14 clades whose divergence times were newly estimated, we first used maximum likelihood (ML) to conduct nonparametric bootstrap analyses for each locus in RAxML v.7.0.4 [11] and then removed those species that were a source of significant incongruence between different locus trees based on the threshold of bootstrap value >70% [12]. For the final concatenated datasets, we performed RAxML analyses with the GTR + G substitution model for each locus, in which nodal supports were assessed using the fast bootstrap option with 1000 replicates. New time-calibrated phylogenies were inferred in BEAST v.1.10.4 [13] using the optimal RAxML tree as the starting tree. Time calibration was done using fossil constraints or secondary calibration points, depending on the information available for each clade. For details of the calibrations used for the 14 clades, see the section on ***Phylogenetic analysis and calibration details for the 14 clades*** below. Under the uncorrelated relaxed molecular clock model, birth-death tree prior, and GTR + G model for each locus, all BEAST analyses were run for 50 million generations, sampling every 10,000 generations. Convergence and the adequate effective sample size values (> 200) were checked using Tracer v.1.7.1 [14]. After discarding the first 25% of trees as burn-in, the maximum clade credibility (MCC) tree with median ages and 95% highest posterior density (HPD) intervals for nodes was generated using TreeAnnotator v.1.10.4 (part of the BEAST package).

**Phylogenetic analysis and calibration details for the 14 clades**

**(1) Hydrangeeae (Hydrangeaceae, Cornales)**

One nuclear (ITS: 659 bp) and four plastid (*ndhA*: 1168 bp, *rpl32-ndhF*: 1630 bp, *trnL-rpl32*: 1362 bp, *trnV-ndhC*: 1420 bp) DNA regions were used to construct a dated phylogeny for Hydrangeeae. We used two secondary calibration points, using ages estimated in a broad study of Cornales [15]: 59.2 Ma (standard deviation [SD] = 9.6) for the stem group age of Hydrangeaceae and 82.6 Ma (SD = 6.0) for the root age. Our divergence time estimate suggests a crown group age of 31.53 Ma (95% HPD: 23.54−39.66) for Hydrangeeae, which coincides with the time estimate of Raman et al. [16] (27.57 Ma; 95% HPD: 20.74−35.75).

**(2) Stachyuraceae (Crossosomatales)**

Seven plastid DNA regions (*matK*: 1515 bp, *ndhF*: 2084 bp, *psbA-trnH*: 469 bp, *rbcL*: 1362 bp, *rps16*: 794 bp, *trnL-F*: 907 bp, *trnS-trnG*: 1305 bp) were used to construct a dated phylogeny for Stachyuraceae. We used two secondary calibration points, using ages estimated in a broad study of Crossosomatales [17]: 54.29 Ma (SD = 3.0) and 6.85 Ma (SD = 3.2) for the stem and crown group ages of Stachyuraceae, respectively. Our divergence time estimate suggests a crown group age of 9.02 Ma (95% HPD: 3.59−14.3) for Stachyuraceae, which coincides with the time estimate of Feng et al. [18] (8.83 Ma; 95% HPD: 5.2−17.1).

**(3) Theaceae (Ericales)**

The reduced dataset of Yan et al. [19] containing plastid coding regions (CDS) and nuclear ribosomal DNA (nrDNA; 18S-ITS1-5.8S-ITS2-26S) was used to construct a dated phylogeny for Theaceae. Following the results of Yan et al. [19], we used three secondary calibration points: 66.4 Ma (SD = 3.3) for the crown group age of Theaceae, 25.4 Ma (SD = 2.3) for the crown group age of Theeae, and 92.1 Ma (SD = 4.0) for the root age. We also used the fossil of *Schima kwangsiensis* from the Late Oligocene (23.03−27.82 Ma) [20] to give a lower bound of 23.03 Ma for the stem group age of *Schima*. Our divergence time estimate suggests a crown group age of 28.4 Ma (95% HPD: 23.85−32.02) for Gordonieae, which coincides with the result of Yu et al. [21] (26.2 Ma; 95% HPD: 23.3–32.2).

**(4) *Sophora* (Fabaceae, Fabales)**

Four plastid DNA regions (*matK*: 756 bp, *rbcL*: 1235 bp, *rpl32-trnL*: 467 bp, *trnL-F*: 1015 bp) were used to construct a dated phylogeny for *Sophora*. Based on the results of Liao et al. [22], we used two secondary calibration points: 44.24 Ma (SD = 4.5) and 36.99 Ma (SD = 4.6) for the stem and crown group ages of *Sophora*, respectively.

**(5) Juglandaceae (Fagales)**

One nuclear (ITS: 700 bp) and seven plastid (*atpB-rbcL*: 767 bp, *matK*: 1254 bp, *psbA-trnH*: 254 bp, *rpl32-trnH*: 509 bp, *rps16*: 1131 bp, *trnL-F*: 398 bp, *trnS-trnG*: 1024 bp) DNA regions were used to construct a dated phylogeny for Juglandaceae. Following Yan et al. [23], we selected four calibration points: 1) the oldest fagalean fossil *Soepadmoa cupulata* from the Turonian (89.8−93.9 Ma) [24] was used to constrain the root age with a lognormal prior distribution (offset = 89.8, SD = 1.0); 2) the fruit fossil *Polyptera manningii* from the Early Paleocene (64.4 Ma) [25,26] was used to give a lower bound of 64.4 Ma for the crown group age of Juglandoideae; 3) the fruit fossil *Cyclocarya brownii* from the Paleocene (56−61.6 Ma) was used to give a lower bound of 56 Ma for the stem group age of *Cyclocarya*; and 4) the fruit fossil *Pterocarya occidentalis* from the Oligocene (23.03−33.9 Ma) [27] was used to give a lower bound of 23.03 Ma for the crown group age of *Pterocarya*.

**(6) *Ligustrum* (Oleaceae, Lamiales)**

The reduced plastome dataset of Wang et al. [28] was used to construct a dated phylogeny for *Ligustrum*. Based on the results of Wang et al. [28], we selected two secondary calibration points: 27.54 Ma (SD = 2.5) for the crown group age of *Ligustrum* and 25.52 Ma (SD = 2.0) for the crown group age of *Ligustrum* sect. *Subdrupacea*. In addition, the leaf fossil *Ligustrum miovulgare* from the Middle Miocene (Serravallian; 11.63−13.82 Ma) close to extant *L*. *vulgare* [29] was used to give a lower bound of 11.63 Ma for the stem group age of *L*. *vulgare*.

**(7) Calycanthaceae (Laurales)**

Nine plastid DNA regions (*atpB*: 1463 bp, *atpB-rbcL*: 858 bp, *matK*: 1563 bp, *ndhF*: 1957 bp, *rbcL*: 1371 bp, *rpl16*: 1481 bp, *rpoC2*: 4167 bp, *trnL-F*: 1109 bp, *trnT-trnL*: 861 bp) were used to construct a dated phylogeny for Calycanthaceae. Six fossil calibration points were selected: 1) the Miocene fossil fruits assigned to *Calycanthus* [30,31] were used to give a lower bound of 16 Ma for the crown group age of *Calycanthus*; 2) the fossil *Jerseyanthus calycanthoides* from the Coniacian sister to extant *Calycanthus* [32,33] was used to give a lower bound of 86.3 Ma for the crown group age of Calycanthoideae; 3) the fossil *Virginianthus calycanthoides* from the Middle Albian [33,34] was used to give a lower bound of 107.7 Ma for the stem group age of Calycanthaceae; 4) the fossil *Monimiophyllum callidentatum* from the Eocene [35] was used to give a lower bound of 52.22 Ma for the crown group age of Monimiaceae; 5) the fossil *Illigera eocenica* from the Middle Eocene [36,37] was used to give a lower bound of 41.2 Ma for the crown group age of Hernandiaceae; and 6) the earliest pollen of Atherospermataceae from the Late Cretaceous [38] was used to give a lower bound of 86 Ma for the stem group age of Atherospermataceae. The root age was constrained to 132.25 Ma (SD = 4.0) based on the result of Li et al. [39]. Our divergence time estimate suggests a crown group age of 103.28 Ma (95% HPD: 92.66−114.24) for Calycanthaceae, which coincides with the time estimate of Ramírez-Barahona et al. [40] (107.85 Ma; 95% HPD: 94.71−122.98).

**(8) *Litsea* complex (Lauraceae, Laurales)**

The reduced dataset of Qin et al. [41] containing plastid CDS and rRNA genes was used to construct a dated phylogeny for the *Litsea* complex. Following the results of Qin et al. [41], we used two secondary calibration points: 52.65 Ma (SD = 1.7) and 46.73 Ma (SD = 2.0) for the stem and crown group ages of the *Litsea* complex, respectively.

**(9) Elaeagnaceae (Rosales)**

One nuclear (ITS: 707 bp) and six plastid (*matK*: 1539 bp, *ndhF*: 2229 bp, *psbA-trnH*: 458 bp, *rbcL*: 1398 bp, *rpoC2*: 4179 bp, *trnL-F*: 1093 bp) DNA regions were used to construct a dated phylogeny for Elaeagnaceae. Based on the results of Onstein and Linder [42], we used two secondary calibration points: 83.49 Ma (SD = 7.2) for the stem group age of Elaeagnaceae and 98.7 Ma (SD = 5.2) for the root age. Our divergence time estimate suggests a crown group age of 42.37 Ma (95% HPD: 28.24−55.72) for Elaeagnaceae, which coincides with the time estimate of Jia and Bartish [43] (40.62 Ma; 95% HPD: 36.94−44.04).

**(10) Rhamneae (Rhamnaceae, Rosales)**

The reduced dataset of Tian et al. [44] containing 89 low-copy nuclear loci was used to construct a dated phylogeny for Rhamneae. Following Hauenschild et al. [45], two fossil calibration points were used with lognormal prior distributions: 1) the fossil *Frangula hordwellensis* from the Late Eocene (33.9−41.2 Ma) [46] was used to constrain the stem group age of *Frangula* with an offset of 33.9 Ma (SD = 1.0); 2) the fossils *Rhamnus salicifolius* [47] and *Coahuilanthus belindae* [48] from the Late Cretaceous were used to constrain the combined crown group age of Rhamneae and Maesopsideae with an offset of 70 Ma (SD = 1.0). The root age was constrained to 75.03 Ma (SD = 1.0) based on a broad study of Rhamnaceae [44].

**(11) *Cotoneaster* (Rosaceae, Rosales)**

The whole plastome dataset (157,974 bp) was used to construct a dated phylogeny for *Cotoneaster*. Based on the results of Meng et al. [49], we used two secondary calibration points: 42.67 Ma (SD = 2.5) and 20.49 Ma (SD = 1.5) for the stem and crown group ages of *Cotoneaster*, respectively. In addition, the fossil *Photinia pageae* from the Middle Eocene (37.71−47.8 Ma) [50] was used to give a lower bound of 37.71 Ma for the stem group age of *Photinia*.

**(12) Ulmaceae (Rosales)**

The nrDNA (18S-ITS1-5.8S-ITS2-26S: 4197 bp) and four plastid DNA regions (*matK*: 1539 bp, *psbA-trnH*: 1291 bp, *rbcL*: 1428 bp, *trnL-F*: 1251 bp) were used to construct a dated phylogeny for Ulmaceae. Following Zhang et al. [51], we selected four fossil calibration points: 1) the Early Cenozoic fossils attributed to *Zelkova*, which resemble extant *Z. schneideriana* [52], were used to give a lower bound of 50 Ma for the crown group age of *Zelkova*; 2) the unequivocal leaf fossils *Ulmus furcinervis* from the Paleocene [53] were used to give a lower bound of 56 Ma for the stem group age of *Ulmus*; 3) the fossils of *U.* sect. *Chaetoptelea* [54,55] were used to give a lower bound of 47.8 Ma for the stem group age of Sect. *Chaetoptelea* (*U. alata*); and 4) the fossils of *U.* sect. *Ulmus* [56] and *Ulmus tenuinervis* of *U*. sect. *Microptelea* [57,58] from the Early Oligocene were used to give a lower bound of 28.1 Ma for the combined crown group age of sections *Ulmus* and *Microptelea*. The root age was constrained to 85.39 Ma (SD = 5.0) based on the result of Zhang et al. [51]. Our divergence time estimate suggests a crown group age of 51.33 Ma (95% HPD: 38.23−65.96) for *Ulmus* subg. *Ulmus*, which coincides with the time estimate of Zhang et al. [51] (52.75 Ma; 95% HPD: 36.65−65.76).

**(13) *Acer* (Sapindaceae, Sapindales)**

The whole plastome dataset (157,043 bp) was used to construct a dated phylogeny for *Acer*. Following Renner et al. [59] and Li et al. [60], we used the fruit fossil *Dipteronia* *brownii* from the Paleocene (60−63 Ma) [61] to constrain the stem group age of *Dipteronia* (the root age) with an exponential prior distribution (offset = 60, mean = 1). In addition, the leaf fossil *Acer dettermani* from the Middle Eocene [62] assignable to *Acer* sect. *Macrantha* [63] was used to give a lower bound of 43 Ma for the stem group age of *A*. sect. *Macrantha* following Areces-Berazain et al. [64].

**(14) Altingiaceae-Hamamelidaceae clade (containing Altingiaceae, Cercidiphyllaceae, Daphniphyllaceae, and Hamamelidaceae, Saxifragales)**

The dataset of Xiang et al. [65] containing seven DNA regions (ITS, *atpB*, *atpB-rbcL*, *matK*, *psbA-trnH*, *rbcL*, *trnL-F*) was used to construct a dated phylogeny for the Altingiaceae-Hamamelidaceae clade. Nine fossil calibration points were selected: 1) the fossil *Rhodoleia cretacea* from the Late Maastrichtian (66−71 Ma) [66] was used to give a lower bound of 66 Ma for the stem group age of *Rhodoleia*; 2) the fossil *Mytilaria boglei* from the Middle Eocene (37.71−47.8 Ma) [67] was used to give a lower bound of 37.71 Ma for the stem group age of *Mytilaria*; 3) the fossil *Disanthus nipponicus* from the Oligocene (23.03−33.9 Ma) [68] was used to give a lower bound of 23.03 Ma for the stem group age of *Disanthus*; 4) the fossil *Allonia decandra* from the Late Santonian to Early Campanian (83−84 Ma) [69] is a member of the clade containing tribes Loropetaleae and Corylopsideae [70,71] and was used to give a lower bound of 83 Ma for the stem group age of this clade; 5) the fossil *Corylopsis reedae* from the Early Eocene (47.8−50 Ma) [72] was used to give a lower bound of 47.8 Ma for the stem group age of *Corylopsis*; 6) the fossil *Fortunearia kabutoiwana* from the Late Miocene (5.33−11.63 Ma) [73] was used to give a lower bound of 5.33 Ma for the stem group age of *Fortunearia*; 7) the fossil *Archamamelis bivalvis* from the Late Santonian to Early Campanian (83−84 Ma) [74] is sister to *Hamamelis* [70] and was used to give a lower bound of 83 Ma for the stem group age of *Hamamelis*; 8) the fossil *Parrotiopsis shimanskiana* from the Late Paleocene (56−59.2 Ma) [75] was used to give a lower bound of 56 Ma for the stem group age of *Parrotiopsis*; and 9) the fossil of *Shaniodendron viburnifolia* from the Middle Miocene (11.63−15.92 Ma) [76,77] was used to give a lower bound of 11.63 Ma for the stem group age of *Shaniodendron*. The root age was constrained to 125.27 Ma (SD = 1.0) based on the result of Ramírez-Barahona et al. [40].

**Biogeographic and habit analyses**

To assess the evolutionary patterns of evergreen lineages in subtropical East Asia, we conducted biogeographic and habit analyses for each of the 21 clades. Information on distribution and habit of each species was compiled from field expeditions, literature, and online database, including Alabama Plant Atlas (<http://floraofalabama.org/>), Atlas of Living Australia (<https://www.ala.org.au/>), CABI Digital Library (<https://www.cabidigitallibrary.org/>), eFloras (<http://www.efloras.org/>), Encyclopedia of Life ([https://eol.org](https://eol.org/)), Flora of Zimbabwe (<https://www.zimbabweflora.co.zw/>), Flora Malesiana (<https://portal.cybertaxonomy.org/flora-malesiana/>), Global Biodiversity Information Facility (<https://www.gbif.org/>), Global Plants on JSTOR (<https://plants.jstor.org/>), India Biodiversity Portal (<https://indiabiodiversity.org/>), Leon Levy Native Plant Preserve (<https://levypreserve.org/>), North Carolina Extension Gardener Plant Toolbox (<https://plants.ces.ncsu.edu/>), Plants of the World Online (<https://powo.science.kew.org/>), Queensland Government (<https://www.qld.gov.au/>), San Marcos Growers (<https://www.smgrowers.com/>), Southwest Desert Flora (<http://southwestdesertflora.com/>), Smithsonian Tropical Research Institute (<https://panamabiota.org/stri/index.php>), The Institute for Regional Conservation ([https://www.regionalconservation.org](https://www.regionalconservation.org/)), Trees and Shrubs Online (<https://www.treesandshrubsonline.org/>), Tropicos.org. Missouri Botanical Garden ([http://www.tropicos.org](http://www.tropicos.org/)), WikiMili (<https://wikimili.com/>), USDA Plants Database (<https://plants.usda.gov/home>), Useful Temperate Plants (<https://temperate.theferns.info/>), and Useful Tropical Plants (<https://tropical.theferns.info/>).

For biogeographic analyses, we defined six geographic regions: subtropical East Asia, tropical Asia (i.e., South Asia, Southeast Asia and Oceania), temperate Eurasia (including northern Africa), North America, South America, and Africa (including southern Arabian Peninsula). For each clade, the analysis was run without constraining the availability of connections between two regions, and the maximum number of areas in ancestral nodes was set according to the maximum number of regions that extant taxa occupy. Considering the controversy of the founder-event speciation (*J*) parameter [78,79], here we tested three biogeographic models (DEC, DIVALIKE, and BAYAREALIKE) based on weighted Akaike Information Criterion corrected for sample size (AICc_wt) using an ML framework. Under the best-fit model with the highest AICc_wt (Table S6), ancestral ranges for each clade were inferred using BioGeoBEARS [80] implemented in RASP v.4.2 [81].

For habit analyses, we coded two states: evergreen and deciduous. For semi-evergreen or semi-deciduous species, we coded them as both evergreen and deciduous. The ancestral habit for each clade was reconstructed using the Bayesian Binary Markov chain Monte Carlo (BBM) method implemented in RASP v.4.2 [81]. We applied 10 simultaneous chains optimized with the fixed JC + G model for 5,000,000 generations, sampling every 1000 generations.

**Assembly dynamic multi-taxon analysis**

Based on the results of biogeographic and habit analyses, we categorized the species-level events for evergreen lineages in subtropical East Asia on the phylogenetic tree (Fig. S6) into three types: habit transition from deciduous to evergreen, *in situ* diversification, and dispersal into subtropical East Asia. We first extracted credibility intervals of divergence times for nodes at which these three types of events occurred, and then calculated the MTE, MDivE, and MDisE, respectively, by summing up potential transition, *in situ* diversification, or dispersal events over all datasets through time using time slices of 1 Myr. The data were smoothed by calculating mean values using a sliding window approach with a time frame of 5 Myr. Additionally, we extracted dispersal times from different source regions: tropical Asia (11 events) and temperate Eurasia (4 events), and calculated their respective MDisEs using the same approach.

The inflection points in the MTE and MDivE curves were identified using the divisive hierarchical estimation algorithm [82] in the R package ecp v.1.6.2 [83]. The “e.divisive” function was invoked with the following parameters: significance level = 0.01, maximum number of random permutations = 1000, number of change point locations = NULL (to only return statistically significant estimated change points), minimum number of observations between changes = 5 Ma (to avoid inferring excessive changes of small magnitude). For comparison, we also calculated the MDivE and MDisE by summing up potential *in situ* diversification and dispersal events over the datasets of the first five dominant families in East Asian EBLF, i.e., Fagaceae, Lauraceae, Magnoliaceae, Theaceae and Hamamelidaceae [84]. In addition, we depicted the age distribution of stem nodes of evergreen species in subtropical East Asia using raincloud plots, and visualized age estimates for dispersal events out of East Asian evergreen broadleaved forest in the R package ggplot2 v.3.3.2 [85].

**Macroecological analysis**

To explore changes in paleoenvironments that are suitable for the development of East Asian EBLF, we estimated the changes of suitable areas of this biome through time. The climatic parameters were constrained at mean annual temperature = 13–20℃ and aridity index = 0, equating to a rough approximation of a subtropical environment likely to be dominated by East Asian EBLF [84]. The aridity index was set at 1 for regions defined as arid by the Köppen band and 0 for all the other bands [86]. We counted 2° × 2° grid cells from paleoenvironmental reconstruction corresponding approximately to this biome through time, and recorded changes in suitable area. Given that most of aforementioned biological events occurred in the Cenozoic, we compiled landscape data of East Asia since ~66 Ma using the database of Hagen et al. [86].

To evaluate the correlation between evolutionary dynamics of evergreen lineages (MTE, MDivE, and MDisE) and environmental changes over time, we performed generalized least squares (GLS) multiple regression analyses using the “gls” function in the R package nlme v.3.1-152 [87]. The predictor variables included East Asian annual precipitation [88] and global temperature [89]. The significance of each predictor was assessed using *t*-tests based on the robust standard errors estimated by GLS. Given that East Asian annual precipitation was found to be correlated with MTE and MDivE significantly (*p*-value < 0.05) or MDisE marginally significantly (*p*-value < 0.1) and global temperature was not significantly correlated with these three events (Table S2), we further used partial regression plots [90] to visualize the partial effect of precipitation on evolutionary dynamics of evergreen lineages after removing the effect of temperature via residualization in the R package ggplot2 v.3.3.2 [85].

We also tested whether three historical events associated with Asian monsoon climate change have impacted the MTE, MDivE, and MDisE via breakpoint regression analyses using Bayesian inference. These events included northward expansion of Asian monsoon to subtropical southern China (~41 Ma), formation of modern-like Asian monsoon system (~25–23 Ma), and decrease in East Asian precipitation (~3.6–2.6 Ma). The analyses were conducted with the R package mcp v.0.3.4 [91] using the scripts modified from Wootton et al. [92]. The models with 0–3 break points were tested, and parameterized with unconnected intercepts and slope values set to zero. The fits of models were assessed using leave-one-out cross-validation implemented in the R package loo v.2.8.0 [93].

**Fig. S1.** Distribution of the selected 21 clades in the angiosperm order-level tree of life.

**Fig. S2.** Ancestral range and habit reconstructions of the 21 clades investigated.

**Fig. S3.** Transition from deciduous to evergreen habits and *in situ* diversification of evergreen lineages in East Asian evergreen broadleaved forests through time.

**Fig. S4.** *In situ* diversification of evergreen lineages and dispersal from other regions for the first five dominant families in East Asian evergreen broadleaved forests.

**Fig. S5.** Age estimates for dispersal events out of East Asian evergreen broadleaved forests.

**Fig. S6.** Identification of habit transition from deciduous to evergreen, *in situ* diversification, and dispersal events at the species level.

**Table S1.** Sampling information for the selected 21 clades analyzed in this study.

**Table S2.** Results of generalized least squares multiple regression analyses.

**Table S3.** Estimated ages of 48 evergreen lineages/species that transitioned from deciduous lineages in subtropical East Asia.

**Table S4.** Estimated ages and source areas of 15 evergreen lineages in subtropical East Asia that dispersed from other regions.

**Table S5.** Species and GenBank accession numbers for the datasets newly assembled in this study.

**Table S6.** Comparison of the fit of the three biogeographic models.

**Fig. S1**

**Fig. S1.** Distribution of the selected 21 clades in the angiosperm order-level tree of life. The phylogenetic tree is modified from APG IV [94]. The orders and families which the selected 21 clades belong to are in bold.

**Fig. S2a**

**Fig. S2b**

**Fig. S2c**

**Fig. S2d**

**Fig. S2e**

**Fig. S2f**

**Fig. S2g**

**Fig. S2h**

**Fig. S2i**

**Fig. S2j**

**Fig. S2k**

**Fig. S2l**

**Fig. S2m**

**Fig. S2n**

**Fig. S2o**

**Fig. S2p**

**Fig. S2q**

**Fig. S2r**

**Fig. S2s**

**Fig. S2t**

**Fig. S2u**

**Fig. S2.** Ancestral range and habit reconstructions of the 21 clades investigated. (a) Ancestral range (left) and habit (right) reconstructions of Aquifoliaceae. (b) Ancestral range (left) and habit (right) reconstructions of Hydrangeeae. (c) Ancestral range (left) and habit (right) reconstructions of Stachyuraceae. (d) Ancestral range (left) and habit (right) reconstructions of *Viburnum*. (e) Ancestral range (left) and habit (right) reconstructions of *Rhododendron*. (f) Ancestral range (left) and habit (right) reconstructions of Theaceae. (g) Ancestral range (left) and habit (right) reconstructions of *Sophora*. (h) Ancestral range (left) and habit (right) reconstructions of Fagaceae. (i) Ancestral range (left) and habit (right) reconstructions of Juglandaceae. (j) Ancestral range (left) and habit (right) reconstructions of *Ligustrum*. (k) Ancestral range (left) and habit (right) reconstructions of Calycanthaceae. (l) Ancestral range (left) and habit (right) reconstructions of *Litsea* complex. (m) Ancestral range (left) and habit (right) reconstructions of Magnoliaceae. (n) Ancestral range (left) and habit (right) reconstructions of Lardizabalaceae. (o) Ancestral range (left) and habit (right) reconstructions of *Celtis*. (p) Ancestral range (left) and habit (right) reconstructions of Elaeagnaceae. (q) Ancestral range (left) and habit (right) reconstructions of Rhamneae. (r) Ancestral range (left) and habit (right) reconstructions of *Cotoneaster*. (s) Ancestral range (left) and habit (right) reconstructions of Ulmaceae. (t) Ancestral range (left) and habit (right) reconstructions of *Acer*. (u) Ancestral range (left) and habit (right) reconstructions of the Altingiaceae-Hamamelidaceae clade. Pie charts on each node show the relative probabilities of alternative ancestral states. Ancestral ranges were inferred under the best-fit biogeographic model (Table S6) using BioGeoBEARS implemented in RASP. Ancestral habits were inferred using the Bayesian Binary Markov chain Monte Carlo (BBM) method in RASP.

**Fig. S3**

**Fig. S3.** Transition from deciduous to evergreen habits and *in situ* diversification of evergreen lineages in East Asian evergreen broadleaved forests through time.

**Fig. S4**

**Fig. S4.** *In situ* diversification of evergreen lineages and dispersal from other regions for the first five dominant families in East Asian evergreen broadleaved forests. The first five dominant families in this biome include Fagaceae, Lauraceae, Magnoliaceae, Theaceae, and Hamamelidaceae [84].

**Fig. S5**

**Fig. S5.** Age estimates for dispersal events out of East Asian evergreen broadleaved forests. The different sink regions are indicated by colors. Black dots and horizontal bars represent the median ages and 95% confidence intervals, respectively.

**Fig. S6**

**Fig. S6.** Identification of habit transition from deciduous to evergreen, *in situ* diversification, and dispersal events at the species level. Example of two phylogenetic trees showing the ancestral ranges and habits depicted for each node.

**Table S1. Sampling information for the selected 21 clades analyzed in this study.**

|  |  |  |  |  | **Sampled evergreen species in subtropical East Asia** | | **Sampled deciduous species in subtropical East Asia** | |
| --- | --- | --- | --- | --- | --- | --- | --- | --- |
| **Order** | **Family** | **Clade** | **Total species** | **Sampled species** | **Total** | **Endemic** | **Total** | **Endemic** |
| Aquifoliales | Aquifoliaceae | Aquifoliaceae | 500 | 169 (33.8%) | 89 | 58 | 12 | 6 |
| Cornales | Hydrangeaceae | Hydrangeeae | 80 | 48 (60.0%) | 10 | 4 | 29 | 23 |
| Crossosomatales | Stachyuraceae | Stachyuraceae | 8 | 8 (100%) | 2 | 1 | 4 | 2 |
| Dipsacales | Viburnaceae | *Viburnum* | 163 | 153 (93.9%) | 29 | 15 | 45 | 23 |
| Ericales | Ericaceae | *Rhododendron* | 1156 | 200 (17.3%) | 122 | 88 | 10 | 6 |
|  | Theaceae | Theaceae | 368 | 136 (37.0%) | 94 | 66 | 5 | 5 |
| Fabales | Fabaceae | *Sophora* | 70 | 54 (77.1%) | 9 | 5 | 11 | 5 |
| Fagales | Fagaceae | Fagaceae | 730 | 277 (37.9%) | 110 | 56 | 18 | 6 |
|  | Juglandaceae | Juglandaceae | 60 | 51 (85.0%) | 3 | 2 | 16 | 5 |
| Lamiales | Oleaceae | *Ligustrum* | 46 | 33 (71.7%) | 15 | 13 | 7 | 4 |
| Laurales | Calycanthaceae | Calycanthaceae | 10 | 10 (100%) | 4 | 4 | 4 | 1 |
|  | Lauraceae | *Litsea* complex | 400 | 96 (24.0%) | 67 | 34 | 19 | 7 |
| Magnoliales | Magnoliaceae | Magnoliaceae | 227 | 98 (43.2%) | 34 | 11 | 23 | 18 |
| Ranunculales | Lardizabalaceae | Lardizabalaceae | 40 | 23 (57.5%) | 13 | 8 | 5 | 2 |
| Rosales | Cannabaceae | *Celtis* | 73 | 39 (53.4%) | 1 | 0 | 12 | 4 |
|  | Elaeagnaceae | Elaeagnaceae | 60 | 35 (58.3%) | 17 | 14 | 4 | 0 |
|  | Rhamnaceae | Rhamneae | 306 | 178 (58.2%) | 12 | 7 | 42 | 20 |
|  | Rosaceae | *Cotoneaster* | 90 | 65 (72.2%) | 18 | 13 | 25 | 9 |
|  | Ulmaceae | Ulmaceae | 56 | 42 (75.0%) | 1 | 0 | 16 | 10 |
| Sapindales | Sapindaceae | *Acer* | 129 | 101 (78.3%) | 8 | 3 | 53 | 32 |
| Saxifragales | Altingiaceae, Cercidiphyllaceae, Daphniphyllaceae, Hamamelidaceae | Altingiaceae-  Hamamelidaceae clade | 127 | 68 (53.5%) | 22 | 4 | 21 | 18 |

**Table S2. Results of generalized least squares multiple regression analyses.** MTE, maximum number of observed transition events; MDivE, maximum number of observed *in situ* diversification events; MDisE, maximum number of observed dispersal events. The data of East Asian annual precipitation and global temperature are from Farnsworth et al. [88] and Westerhold et al. [89], respectively. Significance levels: * = marginally significant if < 0.1, ** = significant if < 0.05.

| **Predictor** | **Coefficient** | **Standard Error** | ***t*-value** | ***p*-value** |
| --- | --- | --- | --- | --- |
| **MTE** |  |  |  |  |
| East Asian annual precipitation | 0.006 | 0.001 | 4.946 | < 0.001** |
| Global temperature | 0.047 | 0.091 | 0.512 | 0.611 |
| **MDivE** |  |  |  |  |
| East Asian annual precipitation | 0.081 | 0.02 | 4.063 | < 0.001** |
| Global temperature | 1.401 | 1.556 | 0.9 | 0.372 |
| **MDisE** |  |  |  |  |
| East Asian annual precipitation | 0.002 | 0.001 | 1.9195 | 0.063* |
| Global temperature | 0.099 | 0.077 | 1.2849 | 0.208 |

**Table S3. Estimated ages of 48 evergreen lineages/species that transitioned from deciduous lineages in subtropical East Asia.**

|  |  | **Age (Ma)** | | |
| --- | --- | --- | --- | --- |
| **Clade** | **Lineage/species** | **Median** | **Lower** | **Upper** |
| Hydrangeeae | *Hydrangea stylosa-Hydrangea indochinensis* | 7.83 | 4.9 | 10.62 |
|  | *Dichroa yunnanensis-Dichroa hirsuta* | 6.41 | 4.23 | 8.73 |
|  | *Hydrangea lobbii* | 0.57 | 0.07 | 1.2 |
|  | *Pileostegia viburnoides-Decumaria sinensis* | 12.74 | 7.64 | 17.45 |
|  | *Hydrangea integrifolia-Hydrangea jelskii* | 11.82 | 6.71 | 16.17 |
| Stachyuraceae | *Stachyurus obovatus-Stachyurus salicifolius* | 2.54 | 0.14 | 5.91 |
| *Viburnum* | *Viburnum amplificatum-Viburnum lutescens* | 61.65 | 50.6 | 72.16 |
|  | *Viburnum awabuki-Viburnum odoratissimum* | 12.65 | 6.35 | 19.78 |
|  | *Viburnum taitoense-Viburnum corymbiflorum* | 16.22 | 9.97 | 23.33 |
|  | *Viburnum oliganthum* | 1.99 | 0.38 | 4.07 |
|  | *Viburnum punctatum-Viburnum lepidotulum* | 35.21 | 23.07 | 48.56 |
|  | *Viburnum utile* | 4.75 | 1.62 | 8.66 |
|  | *Viburnum congestum* | 6.53 | 2.73 | 11.67 |
|  | *Viburnum rhytidophyllum* | 2.27 | 0.53 | 4.07 |
|  | *Viburnum cylindricum-Viburnum leiocarpum* | 33.1 | 22.42 | 43.09 |
|  | *Viburnum lancifolium-Viburnum japonicum* | 10.81 | 7.14 | 14.5 |
|  | *Viburnum annamensis-Viburnum sempervirens* | 4.77 | 2.2 | 7.36 |
| *Rhododendron* | *Rhododendron latoucheae-Rhododendron vialii* | 33.65 | 27.58 | 41.06 |
|  | *Rhododendron tsoi-Rhododendron strigosum* | 26.32 | 19.52 | 35.49 |
|  | *Rhododendron kawngsiense-Rhododendron hainanense* | 9.58 | 6.11 | 15.01 |
|  | *Rhododendron subflumineum-Rhododendron simsii* | 8.6 | 5.29 | 13.96 |
| Theaceae | *Hartia sinensis-Stewartia cordifolia* | 9.73 | 7.5 | 12.5 |
| *Sophora* | *Sophora franchetiana-Sophora wightii* | 24.86 | 15.05 | 34.56 |
| Fagaceae | *Quercus phillyreoides* | 31.46 | 24.14 | 37.76 |
| Juglandaceae | *Engelhardia spicata-Engelhardia fenzelii* | 89.67 | 78.51 | 101.46 |
|  | *Carya sinensis* | 15.96 | 7.2 | 26.66 |
| *Ligustrum* | *Ligustrum sempervirens* | 20.15 | 14.07 | 25.67 |
|  | *Ligustrum obovatilimbum-Ligustrumquihoui* | 25.38 | 22.31 | 28.44 |
|  | *Ligustrum microphyllum-Ligustrum ibota* | 5.51 | 3.83 | 7.46 |
| Magnoliaceae | *Magnolia chimantensis-Magnolia fraseri* | 93.4 | 76.73 | 108.03 |
| Lardizabalaceae | *Boquila trifoliata-Parvaria elliptica* | 37.99 | 20.78 | 56.55 |
|  | *Akebia quinata-Akebia longeracemosa* | 2.23 | 0.83 | 3.99 |
| *Celtis* | *Celtis timorensis* | 14.21 | 9.01 | 20.72 |
| Elaeagnaceae | *Elaeagnus gonyanthes-Elaeagnus conferta* | 22.72 | 12.7 | 33.89 |
| Rhamneae | *Berchemia annamensis* | 5.45 | 2.49 | 8.1 |
|  | *Rhamnella rubrinervis* | 6.97 | 5.09 | 10 |
|  | *Rhamnus aure* | 8.09 | 5.21 | 10.6 |
| *Cotoneaster* | *Cotoneaster salicifolius-Cotoneaster fulvidus* | 5.75 | 4.22 | 7.56 |
|  | *Cotoneaster rotundifolius* | 10.67 | 7.29 | 13.88 |
|  | *Cotoneaster microphyllus-Cotoneaster vellaeus* | 4.47 | 2.89 | 6.01 |
|  | *Cotoneaster huahongdongensis* | 0.37 | 0.08 | 0.7 |
| Ulmaceae | *Ulmus lanceifolia* | 51.33 | 38.23 | 65.96 |
| *Acer* | *Acer laurinum* | 18.18 | 12.45 | 24.41 |
|  | *Acer laevigatum* | 7.17 | 4.39 | 11.12 |
|  | *Acer fabri* | 5.44 | 3.77 | 6.86 |
|  | *Acer oblongum* | 15.39 | 11.42 | 19.6 |
|  | *Acer paxii-Acer sino-oblongum* | 22.87 | 18.59 | 27.45 |
| Altingiaceae-  Hamamelidaceae clade | *Distylium buxifolium-Sycopsis sinensis* | 19.48 | 13 | 33.15 |

**Table S4.** **Estimated ages and source areas of 15 evergreen lineages in subtropical East Asia that dispersed from other regions.**

|  |  |  | **Age (Ma)** | | |
| --- | --- | --- | --- | --- | --- |
| **Clade** | **Lineage** | **Source area** | **Median** | **Lower** | **Upper** |
| Aquifoliaceae | *Ilex perado-Ilex perryana* | Temperate Eurasia | 0.79 | 0.22 | 1.5 |
|  | *Ilex crenata-Ilex revoluta* | Tropical Asia | 5.1 | 3.83 | 6.48 |
|  | *Ilex manneiensis-Ilex szechwanensis* | Tropical Asia | 1.01 | 0.26 | 1.85 |
| *Rhododendron* | *Rhododendron micranthum-Rhododendron hirsutum* | Temperate Eurasia | 23.56 | 17.56 | 31.61 |
|  | *Rhododendron aureum-Rhododendron yakushimanum* | Temperate Eurasia | 20.4 | 15.47 | 26.89 |
| Fagaceae | *Lithocarpus lepidocarpus-*  *Lithocarpus bennettii* | Tropical Asia | 20.31 | 11.23 | 29.53 |
|  | *Castanopsis inermis-Castanopsis acuminatissima* | Tropical Asia | 10.3 | 2.83 | 20.82 |
| Theaceae | *Polyspora speciosa-Polyspora axillaris* | Tropical Asia | 11.81 | 5.81 | 15.95 |
|  | *Polyspora longicarpa-*  *Gordonia szechuanensis* | Tropical Asia | 2.59 | 1.67 | 3.6 |
|  | *Gordonia tonkinonsis-Gordonia balansae* | Tropical Asia | 5.3 | 3.09 | 7.71 |
| Rhamneae | *Rhamnus triquetra-Rhamnus alaternus* | Temperate Eurasia | 29.16 | 23.14 | 36.04 |
| *Litsea* complex | *Lindera nacusua-Lindera communis* | Tropical Asia | 5.47 | 2.88 | 8.55 |
| Magnoliaceae | *Magnolia conifera-Magnolia fordiana* var. *calca* | Tropical Asia | 0.64 | 0.28 | 1.5 |
|  | *Magnolia figo-Magnolia odora* | Tropical Asia | 6.23 | 4.42 | 8.35 |
|  | *Magnolia martini-Magnolia ernestii* | Tropical Asia | 7.04 | 5.19 | 10.14 |

**Table S5.** **Species and GenBank accession numbers for the datasets newly assembled in this study.**

**Hydrangeeae (Hydrangeaceae, Cornales)**

| **Species** | **ITS** | ***ndhA*** | ***rpl32-ndhF*** | ***trnL-rpl32*** | ***trnV-ndhC*** |
| --- | --- | --- | --- | --- | --- |
| *Broussaisia arguta* | LN830391 | LN830487 | LN830562 | LN830637 | LN830343 |
| *Cardiandra alternifolia* | LN830369 | LN830445 | LN830520 | LN830595 | LN830301 |
| *Cardiandra densifolia* | - | NC_044804 | NC_044804 | NC_044804 | NC_044804 |
| *Cardiandra moellendorffii* | LN830394 | LN830468 | LN830543 | LN830618 | LN830324 |
| *Decumaria barbara* | LN830372 | HE983586 | HE983546 | HE983566 | HE983426 |
| *Decumaria sinensis* | - | OR268910 | OR268910 | OR268910 | OR268910 |
| *Deinanthe bifida* | LN830371 | LN830448 | LN830523 | LN830598 | LN830304 |
| *Deinanthe caerulea* | LN830389 | LN830466 | LN830541 | LN830616 | LN830322 |
| *Dichroa daimingshanensis* | - | NC_084220 | NC_084220 | NC_084220 | NC_084220 |
| *Dichroa febrifuga* | LN830400 | HE983587 | HE983547 | HE983567 | HE983427 |
| *Dichroa hirsuta* | LN830357 | LN830431 | LN830506 | LN830581 | LN830287 |
| *Dichroa versicolor* | LN830374 | LN830451 | LN830526 | LN830601 | LN830307 |
| *Dichroa yunnanensis* | LN830375 | LN830452 | LN830527 | LN830602 | LN830308 |
| *Hydrangea angustipetala* | LN830382 | HE983588 | HE983548 | HE983568 | HE983428 |
| *Hydrangea angustipetala* f. *macrosepala* | LN830388 | LN830465 | LN830540 | LN830615 | LN830321 |
| *Hydrangea anomala* | LN830402 | LN830481 | LN830556 | LN830631 | LN830337 |
| *Hydrangea arborescens* | - | NC_084224 | NC_084224 | NC_084224 | NC_084224 |
| *Hydrangea aspera* | LN830350 | LN830421 | LN830496 | LN830571 | LN830277 |
| *Hydrangea chinensis* | LN830356 | LN830427 | LN830502 | LN830577 | LN830283 |
| *Hydrangea chungii* | LN830398 | LN830473 | LN830548 | LN830623 | LN830329 |
| *Hydrangea davidii* | - | NC_050783 | NC_050783 | NC_050783 | NC_050783 |
| *Hydrangea formosana* | - | NC_084219 | NC_084219 | NC_084219 | NC_084219 |
| *Hydrangea heteromalla* | LN830346 | LN830415 | LN830490 | LN830565 | LN830271 |
| *Hydrangea hirta* | LN830365 | LN830439 | LN830514 | LN830589 | LN830295 |
| *Hydrangea indochinensis* | LN830384 | LN830462 | LN830537 | LN830612 | LN830318 |
| *Hydrangea integrifolia* | LN830354 | LN830425 | LN830500 | LN830575 | LN830281 |
| *Hydrangea involucrata* | - | HE983593 | HE983553 | HE983573 | HE983433 |
| *Hydrangea jelskii* | - | LN830478 | LN830553 | LN830628 | LN830334 |
| *Hydrangea lobbii* | - | LN830428 | LN830503 | LN830578 | LN830284 |
| *Hydrangea luteovenosa* | LN830380 | HE983594 | HE983554 | HE983574 | HE983434 |
| *Hydrangea macrophylla* | LN830397 | LN830471 | LN830546 | LN830621 | LN830327 |
| *Hydrangea paniculata* | LN830353 | LN830424 | LN830499 | LN830574 | LN830280 |
| *Hydrangea petiolaris* | LN830367 | LN830441 | LN830516 | LN830591 | LN830297 |
| *Hydrangea quercifolia* | - | NC_084225 | NC_084225 | NC_084225 | NC_084225 |
| *Hydrangea robusta* | LN830409 | LN830419 | LN830494 | LN830569 | LN830275 |
| *Hydrangea sargentiana* | LN830411 | HE983598 | HE983558 | HE983578 | HE983438 |
| *Hydrangea scandens* | LN830381 | LN830459 | LN830534 | LN830609 | LN830315 |
| *Hydrangea seemannii* | - | HE983599 | HE983559 | HE983579 | HE983439 |
| *Hydrangea serrata* f. *acuminata* | LN830377 | HE983600 | HE983560 | HE983580 | HE983440 |
| *Hydrangea sikokiana* | LN830370 | LN830446 | LN830521 | LN830596 | LN830302 |
| *Hydrangea strigosa* | LN830412 | LN830463 | LN830538 | LN830613 | LN830319 |
| *Hydrangea stylosa* | LN830386 | LN830472 | LN830547 | LN830622 | LN830328 |
| *Pileostegia viburnoides* | LN830364 | HE983602 | HE983562 | HE983582 | HE983442 |
| *Platycrater arguta* | - | HE983601 | HE983561 | HE983581 | HE983441 |
| *Schizophragma amplum* | - | NC_044807 | NC_044807 | NC_044807 | NC_044807 |
| *Schizophragma hydrangeoides* | - | LN830480 | LN830555 | LN830630 | LN830336 |
| *Schizophragma integrifolium* var. *fauriei* | LN830378 | LN830456 | LN830531 | LN830606 | LN830312 |
| *Schizophragma molle* | LN830383 | HE983603 | HE983563 | HE983583 | HE983443 |
| **Outgroups** |  |  |  |  |  |
| *Carpenteria californica* | LN830399 | LN830476 | LN830551 | LN830626 | LN830332 |
| *Deutzia compacta* | KP120024 | MN380704 | MN380704 | MN380704 | MN380704 |
| *Fendlera rupicola* | - | MN380688 | MN380688 | MN380688 | MN380688 |
| *Jamesia americana* | - | OR268928 | OR268928 | OR268928 | OR268928 |
| *Kirengeshoma palmata* | - | NC_044808 | NC_044808 | NC_044808 | NC_044808 |
| *Loasa tricolor* | - | LN830430 | LN830505 | LN830580 | LN830286 |
| *Philadelphus mexicanus* | LN830393 | LN830489 | LN830564 | LN830639 | LN830345 |
| *Philadelphus pekinensis* | LN830347 | HE983584 | HE983544 | HE983564 | HE983424 |
| *Whipplea modesta* | - | MN380686 | MN380686 | MN380686 | MN380686 |
| *Xylopodia klaprothioides* | - | LN830475 | LN830550 | LN830625 | LN830331 |

**Stachyuraceae (Crossosomatales)**

| **Species** | ***matK*** | ***ndhF*** | ***psbA-trnH*** | ***rbcL*** | ***rps16*** | ***trnL-F*** | ***trnS-trnG*** |
| --- | --- | --- | --- | --- | --- | --- | --- |
| *Stachyurus chinensis* | NC_058889 | NC_058889 | NC_058889 | NC_058889 | NC_058889 | NC_058889 | NC_058889 |
| *Stachyurus cordatulus* | - | DQ307078 | - | - | DQ307093 | DQ307144 | DQ307129 |
| *Stachyurus himalaicus* | NC_068762 | NC_068762 | NC_068762 | NC_068762 | NC_068762 | NC_068762 | NC_068762 |
| *Stachyurus obovatus* | - | DQ307074 | - | DQ307098 | DQ307089 | DQ307140 | DQ307125 |
| *Stachyurus praecox* | DQ443457 | KX999363 | OK416386 | AJ235794 | KX999462 | KX999494 | KX999526 |
| *Stachyurus retusus* | NC_058890 | NC_058890 | NC_058890 | NC_058890 | NC_058890 | NC_058890 | NC_058890 |
| *Stachyurus salicifolius* | - | DQ307069 | - | - | DQ307084 | DQ307135 | DQ307120 |
| *Stachyurus yunnanensis* | NC_058891 | NC_058891 | NC_058891 | NC_058891 | NC_058891 | NC_058891 | NC_058891 |
| **Outgroup** |  |  |  |  |  |  |  |
| *Crossosoma bigelovii* | HQ680694 | KX999358 | KX999391 | KX999424 | KX999457 | KX999489 | KX999521 |

***Sophora* (Fabaceae, Fabales)**

| **Species** | ***matK*** | ***rbcL*** | ***rpl32-trnL*** | ***trnL-F*** |
| --- | --- | --- | --- | --- |
| *Sophora albescens* | ON996772 | ON996697 | ON996635 | ON996849 |
| *Sophora alopecuroides* | ON996775 | ON996700 | ON996638 | ON996852 |
| *Sophora benthamii* | ON996778 | ON996703 | ON996641 | ON996855 |
| *Sophora bhutanica* | - | U74226 | - | - |
| *Sophora bifolia* | OM935764 | OM935764 | OM935764 | OM935764 |
| *Sophora cassioides* | ON996780 | ON996705 | ON996643 | ON996857 |
| *Sophora chathamica* | ON996781 | ON996706 | ON996644 | - |
| *Sophora chrysophylla* | ON996782 | ON996707 | - | ON996858 |
| *Sophora davidii* | ON996786 | ON996709 | ON996645 | ON996862 |
| *Sophora denudata* | ON996789 | ON996712 | ON996647 | ON996863 |
| *Sophora dunnii* | ON996790 | ON996713 | - | ON996864 |
| *Sophora exigua* | OP787197 | - | - | - |
| *Sophora fernandeziana* | ON996792 | ON996715 | ON996648 | ON996865 |
| *Sophora flavescens* var. *flavescens* | ON996793 | ON996716 | ON996649 | ON996866 |
| *Sophora flavescens* var. *galegoides* | ON996794 | ON996717 | - | ON996867 |
| *Sophora franchetiana* | ON996795 | ON996718 | ON996683 | ON996868 |
| *Sophora fraseri* | KU564620 | - | - | - |
| *Sophora fulvida* | ON996796 | ON996719 | ON996650 | - |
| *Sophora godleyi* | ON996797 | ON996720 | ON996651 | - |
| *Sophora griffithii* | ON996798 | ON996721 | ON996652 | ON996869 |
| *Sophora howinsula* | ON996800 | ON996723 | ON996654 | ON996871 |
| *Sophora huamotensis* | OP745446 | - | - | - |
| *Sophora inhambanensis* | ON996801 | ON996724 | ON996655 | ON996872 |
| *Sophora jaubertii* | - | Z70140 | - | - |
| *Sophora koreensis* | MT571487 | MT571487 | MT571487 | MT571487 |
| *Sophora korolkowi* | ON996802 | ON996725 | ON996656 | ON996873 |
| *Sophora lehmannii* | ON996768 | - | - | - |
| *Sophora longicarinata* | ON996803 | ON996726 | ON996657 | - |
| *Sophora macrocarpa* | ON996804 | ON996727 | ON996658 | - |
| *Sophora masafuerana* | ON996806 | ON996729 | ON996660 | ON996874 |
| *Sophora microcarpa* | ON996807 | - | - | - |
| *Sophora microphylla* | ON996808 | ON996730 | ON996662 | ON996875 |
| *Sophora molloyi* | ON996810 | ON996732 | ON996663 | - |
| *Sophora moorcroftiana* | ON996812 | ON996734 | ON996665 | ON996877 |
| *Sophora nuttalliana* | ON996814 | ON996735 | ON996666 | ON996878 |
| *Sophora pachycarpa* | - | - | ON996667 | AF311369 |
| *Sophora* *prazeri* var. *prazeri* | ON996815 | ON996736 | ON996668 | ON996879 |
| *Sophora prazeri* var. *mairei* | ON996818 | ON996739 | ON996671 | ON996882 |
| *Sophora prostrata* | ON996819 | ON996740 | ON996672 | ON996883 |
| *Sophora raivavaensis* | ON996820 | ON996741 | - | ON996884 |
| *Sophora* sp. | ON996821 | ON996742 | - | ON996885 |
| *Sophora stenophylla* | ON996822 | ON996743 | ON996673 | ON996886 |
| *Sophora tetraptera* | ON996824 | ON996745 | ON996675 | - |
| *Sophora tomentosa* subsp*. Bahamensis* | ON996828 | - | - | - |
| *Sophora tomentosa* var. *occidentalis* | ON996829 | ON996749 | ON996679 | ON996890 |
| *Sophora tomentosa* var. *truncata* | - | ON996750 | ON996680 | ON996891 |
| *Sophora tonkinensis* var. *tonkinensis* | ON996831 | ON996752 | ON996685 | ON996893 |
| *Sophora tonkinensis* var. *polyphylla* | ON996832 | ON996753 | ON996686 | ON996894 |
| *Sophora toromiro* | ON996833 | ON996754 | - | ON996895 |
| *Sophora velutina* var. *velutina* | ON996835 | ON996756 | ON996688 | ON996897 |
| *Sophora velutina* var. *cavaleriei* | ON996836 | ON996757 | ON996689 | ON996898 |
| *Sophora wilsonii* | ON996838 | ON996759 | ON996681 | ON996900 |
| *Sophora xanthoantha* | ON996842 | ON996763 | ON996693 | ON996904 |
| *Sophora yunnanensis* | ON996843 | ON996764 | ON996694 | ON996905 |
| **Outgroups** |  |  |  |  |
| *Ammopiptanthus mongolicus* | MK704436 | MK704436 | MK704436 | MK704436 |
| *Ammopiptanthus nanus* | MW349015 | MW349015 | MW349015 | MW349015 |
| *Maackia amurensis* | MN497644 | MN204900 | - | MN497752 |
| *Maackia hupehensis* | MN497646 | MH657935 | - | MN497753 |
| *Salweenia bouffordiana* | MF449303 | MF449303 | MF449303 | MF449303 |
| *Salweenia wardii* | MN497658 | U74251 | - | MN497766 |

**Juglandaceae (Fagales)**

| **Species** | **ITS** | ***atpB-rbcL*** | ***matK*** | ***psbA-trnH*** | ***rpl32-trnH*** | ***rps16*** | ***trnL-F*** | ***trnS-trnG*** |
| --- | --- | --- | --- | --- | --- | --- | --- | --- |
| *Alfaroa costaricensis* | AF303803 | AF303749 | - | - | - | - | AF303776 | - |
| *Alfaroa guanacastensis* | AF303804 | AF303750 | KF419013 | KF201442 | - | KF201409 | AF303777 | - |
| *Alfaroa manningii* | AF303805 | AF303751 | - | - | - | - | AF303778 | - |
| *Alfaroa williamsii* | AF303806 | AF303752 | U92849 | - | - | - | AF303779 | - |
| *Carya aquatica* | KF201301 | KF418855 | KJ772634 | KF201443 | NC_069581 | NC_069581 | KF419055 | NC_069581 |
| *Carya cathayensis* | KF201302 | AF303765 | KP088993 | KF201444 | NC_046572 | NC_046572 | KF419056 | NC_046572 |
| *Carya cordiformis* | AF303820 | AF303766 | HQ593223 | HQ596633 | NC_067508 | NC_067508 | AF303793 | NC_067508 |
| *Carya glabra* | AF174618 | AF303769 | MF349931 | MF348768 | NC_067504 | NC_067504 | AF303796 | NC_067504 |
| *Carya hunanensis* | KF201306 | KF418858 | KF419016 | KF201448 | NC_046435 | NC_046435 | KF201495 | NC_046435 |
| *Carya illinoinensis* | AF174619 | AF303771 | KF419017 | KF201449 | NC_041449 | - | AF303798 | - |
| *Carya kweichowensis* | KF201307 | KF201479 | NC_040864 | KF201450 | NC_040864 | NC_040864 | KF201496 | NC_040864 |
| *Carya laciniosa* | KF201308 | KF418859 | NC_056375 | KF201451 | NC_056375 | NC_056375 | KF419060 | NC_056375 |
| *Carya myristiciformis* | AF303821 | AF303767 | KF419018 | KF201452 | NC_066503 | NC_066503 | AF303794 | NC_066503 |
| *Carya ovata* | AF174620 | AF303768 | U92850 | KF201454 | NC_053774 | NC_053774 | AF303795 | NC_053774 |
| *Carya pallida* | KF201312 | KF201482 | NC_081983 | KF201455 | NC_081983 | NC_081983 | KF201499 | NC_081983 |
| *Carya sinensis* | AF303818 | AF303764 | AY263919 | KF201456 | NC_047200 | NC_047200 | AY147080 | NC_047200 |
| *Carya tomentosa* | EU646134 | AF303770 | AF118039 | KF201458 | NC_067507 | NC_067507 | KF201502 | NC_067507 |
| *Carya tonkinensis* | AF303826 | AF303772 | AF118043 | KF201459 | NC_066504 | NC_066504 | AF303799 | NC_066504 |
| *Cyclocarya paliurus* | MF182368 | AF303763 | AY147098 | KF201460 | NC_034315 | NC_034315 | AY147075 | NC_034315 |
| *Engelhardia apoensis* | MN307517 | - | - | MN307677 | MN307717 | - | MN307557 | MN307637 |
| *Engelhardia borneensis* | OM296107 | - | - | OM304904 | OM304895 | OM304931 | OM304913 | OM304922 |
| *Engelhardia fenzelii* | MN307513 | KF201486 | AY147099 | MN307673 | MN307713 | MN307593 | MN307553 | MN307633 |
| *Engelhardia hainanensis* | MN307515 | NC_068233 | NC_068233 | NC_068233 | NC_068233 | NC_068233 | NC_068233 | NC_068233 |
| *Engelhardia roxburghiana* | KP092756 | NC_046434 | NC_046434 | NC_046434 | NC_046434 | NC_046434 | NC_046434 | NC_046434 |
| *Engelhardia serrata* | EF141010 | EF140992 | KR530782 | KF201463 | OM304894 | OM304930 | EF141001 | OM304921 |
| *Engelhardia spicata* | AF303802 | NC_081985 | NC_081985 | NC_081985 | NC_081985 | NC_081985 | NC_081985 | NC_081985 |
| *Engelhardia villosa* | MN307511 | - | - | MN307671 | MN307711 | MN307591 | MN307551 | MN307631 |
| *Juglans ailantifolia* | FJ043008 | AY293314 | AF118024 | AY293335 | NC_046433 | NC_046433 | KF419062 | NC_046433 |
| *Juglans australis* | AF179568 | AY293319 | AF118025 | AY293343 | - | - | - | - |
| *Juglans californica* | AF338474 | - | AF118027 | AY293331 | - | - | - | - |
| *Juglans cinerea* | MF182366 | AY293318 | AF118029 | AY293347 | NC_035960 | NC_035960 | AF303786 | NC_035960 |
| *Juglans hindsii* | MF182369 | AY293326 | AF118031 | AY293330 | NC_035965 | NC_035965 | NC_035965 | NC_035965 |
| *Juglans jamaicensis* | FJ043017 | - | KJ012649 | KJ426790 | - | - | - | - |
| *Juglans major* | AF338484 | AY293325 | AF118032 | AY293338 | NC_035966 | NC_035966 | NC_035966 | NC_035966 |
| *Juglans mandshurica* | MF182374 | AY293315 | AF118033 | AY293337 | NC_033892 | NC_033892 | AY147077 | NC_033892 |
| *Juglans microcarpa* | AF338488 | AY293324 | AF118034 | AY293332 | NC_046432 | NC_046432 | AF303784 | NC_046432 |
| *Juglans mollis* | FJ043020 | AY293329 | - | AY293340 | - | - | - | - |
| *Juglans neotropica* | FJ043022 | AY293321 | AF118035 | AY293341 | - | - | - | - |
| *Juglans nigra* | AF338492 | AY293327 | AF118036 | AY293339 | NC_035967 | NC_035967 | AF303783 | NC_035967 |
| *Juglans olanchana* | FJ043026 | AY293316 | AF118037 | AY293333 | - | - | - | - |
| *Juglans regia* | HE574846 | AY293322 | AF118038 | HE966671 | NC_028617 | NC_028617 | AF399881 | NC_028617 |
| *Juglans sigillata* | MF182371 | AY293317 | KX526663 | AY293346 | NC_031373 | NC_031373 | MF167465 | NC_031373 |
| *Oreomunnea mexicana* | EF141013 | EF140995 | KF419014 | KF201468 | NC_081986 | NC_081986 | EF141003 | NC_081986 |
| *Oreomunnea pterocarpa* | EF141014 | EF140996 | - | - | - | - | EF141005 | - |
| *Platycarya strobilacea* | AF303808 | AF303754 | NC_035413 | JF708215 | NC_035413 | NC_035413 | NC_035413 | NC_035413 |
| *Pterocarya fraxinifolia* | KT934647 | ON380920 | KF419019 | ON380921 | ON380920 | ON380920 | KT456200 | ON380920 |
| *Pterocarya hupehensis* | KF214260 | ON380921 | AY263918 | KF201471 | ON380921 | ON380921 | AY147079 | ON380921 |
| *Pterocarya macroptera* | AF303814 | AF303760 | ON375425 | KF201470 | ON375425 | ON375425 | KT455834 | ON375425 |
| *Pterocarya stenoptera* | KF994030 | AY293313 | AF118042 | AY293336 | ON375424 | ON375424 | KF419065 | ON375424 |
| *Pterocarya tonkinensis* | KF201322 | KF418862 | AF118043 | KF201473 | ON380924 | ON380924 | KT455828 | ON380924 |
| *Rhoiptelea chiliantha* | AF303800 | AF303746 | U92852 | KF201474 | - | MT701585 | AY147081 | MT701585 |

**Calycanthaceae (Laurales)**

| **Species** | ***atpB*** | ***atpB-rbcL*** | ***matK*** | ***ndhF*** | ***rbcL*** | ***rpl16*** | ***rpoC2*** | ***trnL-trnF*** | ***trnT-trnL*** |
| --- | --- | --- | --- | --- | --- | --- | --- | --- | --- |
| *Calycanthus chinensis* | MG561304 | MG561304 | MG561304 | MG561304 | MG561304 | MG561304 | MG561304 | MG561304 | MG561304 |
| *Calycanthus floridus* | NC_004993 | NC_004993 | NC_004993 | NC_004993 | NC_004993 | NC_004993 | NC_004993 | NC_004993 | NC_004993 |
| *Calycanthus occidentalis* | KX525791 | AF127605 | KX526618 | KX526863 | KX527068 | AF127250 | - | DQ490944 | AF129017 |
| *Chimonanthus campanulatus* | MW166216 | MW166216 | MW166216 | MW166216 | MW166216 | MW166216 | MW166216 | MW166216 | MW166216 |
| *Chimonanthus grammatus* | MW166217 | MW166217 | MW166217 | MW166217 | MW166217 | MW166217 | MW166217 | MW166217 | MW166217 |
| *Chimonanthus nitens* | MW166220 | MW166220 | MW166220 | MW166220 | MW166220 | MW166220 | MW166220 | MW166220 | MW166220 |
| *Chimonanthus praecox* | MT859152 | MT859152 | MT859152 | MT859152 | MT859152 | MT859152 | MT859152 | MT859152 | MT859152 |
| *Chimonanthus salicifolius* | MW166218 | MW166218 | MW166218 | MW166218 | MW166218 | MW166218 | MW166218 | MW166218 | MW166218 |
| *Chimonanthus zhejiangensis* | MW166219 | MW166219 | MW166219 | MW166219 | MW166219 | MW166219 | MW166219 | MW166219 | MW166219 |
| *Idiospermum australiense* | MH377056 | MH377056 | MH377056 | MH377056 | MH377056 | MH377056 | MH377056 | MH377056 | MH377056 |
| **Outgroups** |  |  |  |  |  |  |  |  |  |
| *Atherosperma moschatum* | AF197604 | AF127603 | DQ401343 | JF268468 | AF121362 | AF127249 | DQ407100 | KF586722 | AF129013 |
| *Cassytha filiformis* | MT621631 | MT621631 | MT621631 | - | MT621631 | MT621631 | MT621631 | MT621631 | MT621631 |
| *Cryptocarya yunnanensis* | MT621600 | MT621600 | MT621600 | MT621600 | MT621600 | MT621600 | MT621600 | MT621600 | MT621600 |
| *Doryphora sassafras* | AF293858 | AF127609 | AF542568 | AY218165 | KF586649 | AF127252 | DQ407112 | KF586660 | AF129023 |
| *Glossocalyx longicuspis* | - | AF127611 | - | - | AF040666 | AF127452 | - | AF012405 | - |
| *Gomortega nitida* | - | AF127612 | - | - | - | AF127260 | - | AF012404 | AF264020 |
| *Gyrocarpus americanus* | AJ235487 | AF127613 | DQ401370 | - | KF381159 | AF127261 | DQ407164 | AF012398 | GQ131095 |
| *Hedycarya arborea* | AJ235490 | HE651085 | AM396509 | AY394738 | L12648 | HE651038 | DQ407161 | AM397149 | - |
| *Illigera grandiflora* | MW755975 | MW755975 | MW755975 | MW755975 | MW755975 | - | MW755975 | MW755975 | MW755975 |
| *Myristica teysmannii* | OQ476656 | OQ476656 | OQ476656 | OQ476656 | OQ476656 | OQ476656 | OQ476656 | OQ476656 | OQ476656 |
| *Persea chienkweiensis* | MZ433389 | MZ433389 | MZ433389 | MZ433389 | MZ433389 | MZ433389 | MZ433389 | MZ433389 | MZ433389 |
| *Peumus boldus* | AF209650 | AF127627 | AJ247183 | - | AF040664 | AF127454 | DQ407162 | AF012403 | AF129041 |
| *Siparuna guianensis* | - | - | GQ982092 | - | MG718360 | AF127455 | - | AF289850 | - |

**Elaeagnaceae (Rosales)**

| **Species** | **ITS** | ***matK*** | ***ndhF*** | ***psbA-trnH*** | ***rbcL*** | ***rpoC2*** | ***trnL-F*** |
| --- | --- | --- | --- | --- | --- | --- | --- |
| *Elaeagnus angustifolia* | AF440256 | NC_040992 | NC_040992 | NC_040992 | NC_040992 | NC_040992 | NC_040992 |
| *Elaeagnus bockii* | AF440258 | MW800930 | MW800930 | MW800930 | MW800930 | MW800930 | MW800930 |
| *Elaeagnus caudata* | MW219727 | - | - | - | - | - | - |
| *Elaeagnus commutata* | MG235000 | KX676844 | - | - | JX848456 | - | HM590275 |
| *Elaeagnus conferta* | HG004840 | MK404307 | MK404307 | MK404307 | MK404307 | MK404307 | MK404307 |
| *Elaeagnus difficilis* | - | MW800993 | MW800993 | MW800993 | MW800993 | MW800993 | MW800993 |
| *Elaeagnus formosana* | - | - | - | AB244029 | - | - | - |
| *Elaeagnus glabra* | JQ062499 | LC522137 | LC522137 | LC522137 | LC522137 | LC522137 | LC522137 |
| *Elaeagnus gonyanthes* | KP093086 | KP093899 | - | KP095803 | KP094842 | - | - |
| *Elaeagnus grandifolia* | - | - | - | - | MT384925 | - | - |
| *Elaeagnus henryi* | MH711692 | MZ846204 | MZ846204 | MZ846204 | MZ846204 | MZ846204 | MZ846204 |
| *Elaeagnus lanceolata* | MH711807 | MW800994 | MW800994 | MW800994 | MW800994 | MW800994 | MW800994 |
| *Elaeagnus loureirii* | MH270486 | MH394425 | MH394425 | MH394425 | MH394425 | MH394425 | MH394425 |
| *Elaeagnus macrophylla* | OQ718949 | NC_028066 | NC_028066 | NC_028066 | NC_028066 | NC_028066 | NC_028066 |
| *Elaeagnus matsunoana* | - | LC626322 | - | - | LC626312 | - | - |
| *Elaeagnus mollis* | MH711769 | NC_036932 | NC_036932 | NC_036932 | NC_036932 | NC_036932 | NC_036932 |
| *Elaeagnus montana* | - | LC618896 | - | LC618914 | LC618873 | - | - |
| *Elaeagnus moorcroftii* | EF423362 | NC_086734 | NC_086734 | NC_086734 | NC_086734 | NC_086734 | NC_086734 |
| *Elaeagnus multiflora* | MH808992 | LC522136 | LC522136 | LC522136 | LC522136 | LC522136 | LC522136 |
| *Elaeagnus oldhamii* | GQ396675 | OQ948125 | OQ948125 | OQ948125 | OQ948125 | OQ948125 | OQ948125 |
| *Elaeagnus oxycarpa* | OR678288 | OR738180 | - | - | OR738045 | - | - |
| *Elaeagnus pungens* | MN579519 | NC_058887 | NC_058887 | NC_058887 | NC_058887 | NC_058887 | NC_058887 |
| *Elaeagnus rotundata* | - | LC679861 | - | OK417289 | - | - | AB817355 |
| *Elaeagnus tarokoensis* | - | - | - | - | MT384927 | - | - |
| *Elaeagnus thunbergii* | - | MT384563 | MT384827 | - | MT384926 | - | - |
| *Elaeagnus umbellata* | JF980300 | MN599114 | MN599114 | MN599114 | MN599114 | MN599114 | MN599114 |
| *Hippophae goniocarpa* | AF440255 | MW791524 | MW791524 | MW791524 | MW791524 | MW791524 | MW791524 |
| *Hippophae gyantsensis* | AF440247 | MK552375 | MK552375 | MK552375 | MK552375 | MK552375 | MK552375 |
| *Hippophae litangensis* | KJ844015 | - | - | KJ854986 | - | - | DQ838722 |
| *Hippophae neurocarpa* | JN613500 | NC_047483 | NC_047483 | NC_047483 | NC_047483 | NC_047483 | NC_047483 |
| *Hippophae rhamnoides* | - | NC_035548 | NC_035548 | NC_035548 | NC_035548 | NC_035548 | NC_035548 |
| *Hippophae salicifolia* | FN687765 | NC_056188 | NC_056188 | NC_056188 | NC_056188 | NC_056188 | NC_056188 |
| *Hippophae tibetana* | FN687767 | MN643620 | MN643620 | MN643620 | MN643620 | MN643620 | MN643620 |
| *Shepherdia argentea* | JQ517289 | KX677576 | - | KT167371 | AJ225787 | - | AJ225794 |
| *Shepherdia canadensis* | JQ517290 | JN966666 | - | - | U17039 | - | GQ245524 |
| **Outgroups** |  |  |  |  |  |  |  |
| *Barbeya oleoides* | OP023315 | NC_040984 | NC_040984 | NC_040984 | NC_040984 | NC_040984 | NC_040984 |
| *Dirachma socotrana* | - | JF317423 | JF317442 | - | AJ225789 | JF317502 | AJ225796 |
| *Hovenia trichocarpa* | KR083076 | MT225404 | MT225404 | MT225404 | MT225404 | MT225404 | MT225404 |

***Cotoneaster* (Rosaceae, Rosales)**

| **Species** | **Plastome** |
| --- | --- |
| *Cotoneaster acuminatus* | MK650045 |
| *Cotoneaster acutifolius* | MK638990 |
| *Cotoneaster adpressus* | MK638993 |
| *Cotoneaster affinis* | MK650051 |
| *Cotoneaster argenteus* | MK578683 |
| *Cotoneaster astrophoros* | MK650065 |
| *Cotoneaster brickellii* | MK578681 |
| *Cotoneaster bullatus* | MK614791 |
| *Cotoneaster buxifolius* | MK578684 |
| *Cotoneaster chengkangensis* | MK638992 |
| *Cotoneaster cinerascens* | MK638991 |
| *Cotoneaster cochleatus* | MK524400 |
| *Cotoneaster conspicuus* | MK638987 |
| *Cotoneaster dammeri* | MK605511 |
| *Cotoneaster delavayanus* | MK605518 |
| *Cotoneaster dielsianus* | MK614800 |
| *Cotoneaster foveolatus* | MK650046 |
| *Cotoneaster frigidus* | MK561975 |
| *Cotoneaster fulvidus* | MK614792 |
| *Cotoneaster gamblei* | MK650052 |
| *Cotoneaster glaucophyllus* | MK561976 |
| *Cotoneaster harrysmithii* | MK544859 |
| *Cotoneaster hebephyllus* | MK638988 |
| *Cotoneaster horizontalis* | MK561973 |
| *Cotoneaster huahongdongensis* | MK614796 |
| *Cotoneaster integerrimus* | MK614799 |
| *Cotoneaster lacteus* | MK605517 |
| *Cotoneaster langei* | MK605516 |
| *Cotoneaster leveillei* | MK544857 |
| *Cotoneaster marginatus* | MK605510 |
| *Cotoneaster melanocarpus* | MK561977 |
| *Cotoneaster microphyllus* | MK544856 |
| *Cotoneaster mongolicus* | MK578682 |
| *Cotoneaster morrisonensis* | MK559435 |
| *Cotoneaster moupinensis* | MK614797 |
| *Cotoneaster multiflorus* | MK650060 |
| *Cotoneaster obscurus* | MK614798 |
| *Cotoneaster pannosus* | MK605509 |
| *Cotoneaster perpusillus* | MK638994 |
| *Cotoneaster praecox* | MK638986 |
| *Cotoneaster qungbixiensis* | MK605513 |
| *Cotoneaster reticulatus* | MK650055 |
| *Cotoneaster rockii* | MK605515 |
| *Cotoneaster rotundifolius* | MK650063 |
| *Cotoneaster rubens* | MK614793 |
| *Cotoneaster salicifolius* | KY419943 |
| *Cotoneaster schantungensis* | MK650053 |
| *Cotoneaster serotinus* | MK578685 |
| *Cotoneaster shansiensis* | MK650064 |
| *Cotoneaster sherriffii* | MK614794 |
| *Cotoneaster silvestrii* | MN577894 |
| *Cotoneaster soongoricus* | MK650057 |
| *Cotoneaster sternianus* | MK650050 |
| *Cotoneaster subadpressus* | MK650058 |
| *Cotoneaster submultiflorus* | MK650061 |
| *Cotoneaster taylorii* | MN577872 |
| *Cotoneaster tenuipes* | MK650047 |
| *Cotoneaster turbinatus* | MK650054 |
| *Cotoneaster vandelaarii* | MK544858 |
| *Cotoneaster vellaeus* | MK614795 |
| *Cotoneaster verruculosus* | MK605512 |
| *Cotoneaster vestitus* | MK559433 |
| *Cotoneaster villosulus* | MK650048 |
| *Cotoneaster zabelii* | MK650056 |
| *Cotoneasterfranchetii* | MK638985 |
| **Outgroups** |  |
| *Heteromeles* *arbutifolia* | MK920281 |
| *Heteromeles salicifolia* | KY419965 |
| *Photinia* *beckii* | MN577889 |
| *Photinia integrifolia* | MN577879 |
| *Photinia* *lanuginosa* | MN577890 |
| *Photinia* *lochengensis* | MN577888 |
| *Photinia prionophylla* | MN577891 |
| *Photinia* *prunifolia* | MK920279 |
| *Photinia* *serratifolia* | MN577865 |

**Ulmaceae (Rosales)**

| **Species** | **nrDNA** | ***matK*** | ***psbA-trnH*** | ***rbcL*** | ***trnL-F*** |
| --- | --- | --- | --- | --- | --- |
| *Ampelocera edentula* | MT117100 | MT165902 | MT165902 | MT165902 | MT165902 |
| *Ampelocera hottlei* | MT117101 | MT165903 | MT165903 | MT165903 | MT165903 |
| *Ampelocera longissima* | MT117102 | MT165904 | MT165904 | MT165904 | MT165904 |
| *Ampelocera macrocarpa* | MT117103 | MT165905 | MT165905 | MT165905 | MT165905 |
| *Hemiptelea davidii* | MT117107 | MT165910 | MT165910 | MT165910 | MT165910 |
| *Holoptelea integrifolia* | MT117108 | MT165913 | MT165913 | MT165913 | MT165913 |
| *Phyllostylon rhamnoides* | MT117109 | MT165914 | MT165914 | MT165914 | MT165914 |
| *Planera aquatica* | MT117110 | MT165915 | MT165915 | MT165915 | MT165915 |
| *Ulmus alata* | - | MT165919 | MT165919 | MT165919 | MT165919 |
| *Ulmus americana* | MT117113 | MT165920 | MT165920 | MT165920 | MT165920 |
| *Ulmus bergmanniana* | MT117114 | MT165921 | MT165921 | MT165921 | MT165921 |
| *Ulmus canescens* | - | MT165922 | MT165922 | MT165922 | MT165922 |
| *Ulmus castaneifolia* | MT117115 | MT165923 | MT165923 | MT165923 | MT165923 |
| *Ulmus changii* | MT117116 | MT165924 | MT165924 | MT165924 | MT165924 |
| *Ulmus chenmoui* | MT117117 | MT165925 | MT165925 | MT165925 | MT165925 |
| *Ulmus crassifolia* | MT117118 | MT165926 | MT165926 | MT165926 | MT165926 |
| *Ulmus davidiana* | MT117119 | MT165927 | MT165927 | MT165927 | MT165927 |
| *Ulmus densa* | MT117120 | MT165928 | MT165928 | MT165928 | MT165928 |
| *Ulmus elongata* | - | MT165929 | MT165929 | MT165929 | MT165929 |
| *Ulmus gaussenii* | MT117121 | MT165930 | MT165930 | MT165930 | MT165930 |
| *Ulmus glabra* | MT117122 | MT165931 | MT165931 | MT165931 | MT165931 |
| *Ulmus glaucescens* | MT117123 | MT165932 | MT165932 | MT165932 | MT165932 |
| *Ulmus laciniata* | MT117124 | MT165933 | MT165933 | MT165933 | MT165933 |
| *Ulmus laevis* | MT117125 | MT165934 | MT165935 | MT165936 | MT165937 |
| *Ulmus lamellosa* | MT117126 | MT165935 | MT165935 | MT165935 | MT165935 |
| *Ulmus lanceifolia* | MT117127 | MT165936 | MT165936 | MT165936 | MT165936 |
| *Ulmus macrocarpa* | MT117128 | MT165937 | MT165937 | MT165937 | MT165937 |
| *Ulmus microcarpa* | MT117129 | MT165938 | MT165938 | MT165938 | MT165938 |
| *Ulmus minor* | MT117130 | MT165939 | MT165939 | MT165939 | MT165939 |
| *Ulmus parvifolia* | - | MT165940 | MT165940 | MT165940 | MT165940 |
| *Ulmus prunifolia* | MT117131 | MT165941 | MT165941 | MT165941 | MT165941 |
| *Ulmus pumila* | MT117132 | MT165942 | MT165942 | MT165942 | MT165942 |
| *Ulmus rubra* | MT117133 | MT165943 | MT165943 | MT165943 | MT165943 |
| *Ulmus serotina* | MT117134 | MT165944 | MT165944 | MT165944 | MT165944 |
| *Ulmus szechuanica* | MT117135 | MT165945 | MT165945 | MT165945 | MT165945 |
| *Ulmus thomasii* | MT117136 | MT165946 | MT165946 | MT165946 | MT165946 |
| *Ulmus uyematsui* | MT117137 | MT165947 | MT165947 | MT165947 | MT165947 |
| *Ulmus wallichiana* | - | MT165948 | MT165948 | MT165948 | MT165948 |
| *Zelkova abelicea* | - | MT165949 | MT165949 | MT165949 | MT165949 |
| *Zelkova carpinifolia* | MT117138 | MT165951 | MT165951 | MT165951 | MT165951 |
| *Zelkova schneideriana* | MT117140 | MT165952 | MT165953 | MT165954 | MT165955 |
| *Zelkova serrata* | - | MT165953 | MT165953 | MT165953 | MT165953 |

***Acer* (Sapindaceae, Sapindales)**

| **Species** | **Plastome** |
| --- | --- |
| *Acer acuminatum* | MN864496 |
| *Acer amplum* | MW067026 |
| *Acer argutum* | MW067028 |
| *Acer barbinerve* | OP219550 |
| *Acer buergerianum* | MW067029 |
| *Acer caesium* | MW067030 |
| *Acer calcaratum* | MW067031 |
| *Acer campbellii* | OP219560 |
| *Acer campestre* | MW067033 |
| *Acer cappadocicum* | MK479217 |
| *Acer cappadocicum* subp. *divergens* | MW067034 |
| *Acer cappadocicum* subp. *sinicum* | MW067035 |
| *Acer carpinifolium* | MN864497 |
| *Acer catalpifolium* | NC 041080 |
| *Acer caudatifolium* | MT648756 |
| *Acer ceriferum* | OP219597 |
| *Acer circinatum* | MW067036 |
| *Acer cissifolium* | MW067037 |
| *Acer coriaceifolium* | MW067038 |
| *Acer crataegifolium* | MW067039 |
| *Acer davidii* | NC 030331 |
| *Acer diabolicum* | MW067040 |
| *Acer discolor* | MW067041 |
| *Acer distylum* | MW067042 |
| *Acer elegantulum* | MW067043 |
| *Acer erianthum* | OP219586 |
| *Acer fabri* | MW067044 |
| *Acer fenzelianum* | MK479221 |
| *Acer flabellatum* | MK479222 |
| *Acer glabrum* | MN864498 |
| *Acer granatense* | MW067045 |
| *Acer grandidentatum* | MW067046 |
| *Acer griseum* | MW067047 |
| *Acer grosseri* | OP219530 |
| *Acer henryi* | MW067048 |
| *Acer hyrcanum* | MW067049 |
| *Acer japonicum* | MW067050 |
| *Acer laevigatum* | MF521832 |
| *Acer laurinum* | MW067051 |
| *Acer lobelii* | MW067052 |
| *Acer longipes* | MW067053 |
| *Acer macrophyllum* | MW067054 |
| *Acer mandshuricum* | MW067055 |
| *Acer maximowiczii* | OP219544 |
| *Acer miaotaiense* | NC 030343 |
| *Acer micranthum* | MN864500 |
| *Acer miyabei* | NC 067762 |
| *Acer monspessulanum* | MW067056 |
| *Acer morrisonense* | NC 029371 |
| *Acer negundo* | MN864501 |
| *Acer nikoense* | MN864499 |
| *Acer nipponicum* | MN864502 |
| *Acer oblongum* | MN864503 |
| *Acer obtusatum* | MW067057 |
| *Acer obtusifolium* | MW067058 |
| *Acer olivaceum* | MW067059 |
| *Acer oliverianum* | MW067060 |
| *Acer opalus* | MW067061 |
| *Acer palmatum* | MN864504 |
| *Acer pauciflorum* | MW067062 |
| *Acer paxii* | MW067063 |
| *Acer pectinatum* | MW067064 |
| *Acer pensylvanicum* | MW067065 |
| *Acer pentaphyllum* | MN864505 |
| *Acer pictum* | OP219579 |
| *Acer pilosum* | MN864506 |
| *Acer platanoides* | MN864507 |
| *Acer pseudoplatanus* | MN864508 |
| *Acer pseudosieboldianum* | MW067067 |
| *Acer pubinerve* | OP219549 |
| *Acer pycnanthum* | MW067068 |
| *Acer rubescens* | MW067069 |
| *Acer rubrum* | MN864509 |
| *Acer rufinerve* | MW067070 |
| *Acer saccharinum* | MW067071 |
| *Acer saccharum* | MW067075 |
| *Acer sempervirens* | MW067076 |
| *Acer serrulatum* | MW067077 |
| *Acer shenkanense* | MW067078 |
| *Acer sikkimense* | MW067079 |
| *Acer sino-oblongum* | KY987160 |
| *Acer skutchii* | MW067080 |
| *Acer stachyophyllum* | MW067081 |
| *Acer sterculiaceum* subp. *franchetii* | MW067083 |
| *Acer sterculiaceum* subp. *sterculiaceum* | MN864510 |
| *Acer takesimense* | MW067084 |
| *Acer tataricum* subp. *aidzuense* | MW067087 |
| *Acer tataricum* subp. *ginnala* | MW067085 |
| *Acer tataricum* subp. *tataricum* | MN864511 |
| *Acer tegmentosum* | MW067088 |
| *Acer tenuifolium* | MW067089 |
| *Acer tetramerum* | MK479228 |
| *Acer trautvetteri* | MW067090 |
| *Acer triflorum* | MW067091 |
| *Acer truncatum* | MW067092 |
| *Acer tschonoskii* | OP581036 |
| *Acer tsinglingense* | MW067093 |
| *Acer tutcheri* | MW067094 |
| *Acer velutinum* | MW067095 |
| *Acer wilsonii* | MW067096 |
| *Acer yangbiense* | MT662110 |
| *Dipteronia dyeriana* | NC 031899 |
| *Dipteronia sinensis* | NC 029338 |

**Table S6. Comparison of** **the fit of the three biogeographic models.** Ln*L*, log-likelihood; *d*, rate of range expansion; *e*, rate of range contraction; AICc, corrected Akaike Information Criterion; AICc_wt, weighted AICc. The best model with the highest AICc_wt vaule is in bold.

| **Clade** | **Model** | **Ln*L*** | ***d*** | ***e*** | **AICc** | **AICc_wt** |
| --- | --- | --- | --- | --- | --- | --- |
| Aquifoliaceae | **DEC** | **-294.62** | **0.0099** | **0.0033** | **593.24** | **0.97** |
|  | DIVALIKE | -312.27 | 0.011 | 0.0035 | 628.42 | 2.2E-08 |
|  | BAYAREALIKE | -298.12 | 0.0066 | 0.037 | 600.37 | 2.80E-02 |
| Hydrangeeae | **DEC** | **-89.41** | **0.018** | **0.01** | **183.12** | **0.88** |
|  | DIVALIKE | -91.41 | 0.017 | 0.0079 | 187.14 | 0.12 |
|  | BAYAREALIKE | -100.90 | 0.021 | 0.039 | 206.12 | 9.00E-06 |
| Stachyuraceae | **DEC** | **-5.84** | **0.045** | **1.00E-12** | **18.01** | **0.47** |
|  | DIVALIKE | -6.16 | 0.049 | 1.00E-12 | 18.72 | 0.33 |
|  | BAYAREALIKE | -6.67 | 0.047 | 1.00E-12 | 19.73 | 0.2 |
| *Viburnum* | **DEC** | **-234.72** | **0.01** | **0.001** | **473.53** | **1.00** |
|  | DIVALIKE | -245.24 | 0.01 | 0.0003 | 494.14 | 3.30E-05 |
|  | BAYAREALIKE | -251.57 | 0.011 | 0.02 | 506.11 | 8.30E-08 |
| *Rhododendron* | **DEC** | **-231.35** | **9.40E-05** | **4.70E-06** | **466.16** | **0.73** |
|  | DIVALIKE | -232.12 | 0.0001 | 1.00E-12 | 468.19 | 0.27 |
|  | BAYAREALIKE | -268.54 | 0.0001 | 0.0001 | 540.27 | 6.00E-17 |
| Theaceae | **DEC** | **-215.47** | **0.012** | **1.00E-12** | **434.92** | **0.53** |
|  | DIVALIKE | -224.73 | 0.013 | 4.00E-19 | 453.55 | 4.90E-05 |
|  | BAYAREALIKE | -215.55 | 0.0084 | 0.035 | 435.14 | 0.47 |
| *Sophora* | **DEC** | **-96.46** | **0.014** | **1.00E-12** | **197.13** | **1.00** |
|  | DIVALIKE | -101.94 | 0.014 | 2.00E-09 | 208.24 | 0.0044 |
|  | BAYAREALIKE | -109.36 | 0.022 | 0.052 | 222.82 | 2.70E-06 |
| Fagaceae | **DEC** | **-434.11** | **0.0037** | **0.013** | **872.71** | **0.95** |
|  | DIVALIKE | -498.15 | 0.0058 | 0.0008 | 1000.08 | 1.50E-28 |
|  | BAYAREALIKE | -478.94 | 0.0055 | 0.0013 | 961.92 | 2.90E-20 |
| Juglandaceae | **DEC** | **-77.77** | **0.0042** | **1.00E-12** | **159.81** | **0.99** |
|  | DIVALIKE | -82.28 | 0.0049 | 1.00E-12 | 168.84 | 0.011 |
|  | BAYAREALIKE | -92.25 | 0.0048 | 0.011 | 188.74 | 5.30E-07 |
| *Ligustrum* | DEC | -49.34 | 0.019 | 1.00E-12 | 103.13 | 0.13 |
|  | **DIVALIKE** | **-47.44** | **0.02** | **1.00E-12** | **99.28** | **0.87** |
|  | BAYAREALIKE | -59.95 | 0.023 | 0.023 | 124.35 | 3.10E-06 |
| Calycanthaceae | DEC | -11.23 | 0.0024 | 0.0008 | 28.18 | 0.15 |
|  | **DIVALIKE** | **-9.51** | **0.002** | **1.00E-12** | **24.74** | **0.84** |
|  | BAYAREALIKE | -14.11 | 0.0054 | 0.011 | 33.94 | 0.0084 |
| *Litsea* complex | **DEC** | **-136.23** | **0.02** | **1.00E-12** | **276.54** | **0.91** |
|  | DIVALIKE | -138.53 | 0.018 | 1.00E-12 | 281.20 | 0.087 |
|  | BAYAREALIKE | -146.74 | 0.013 | 0.0065 | 297.53 | 2.40E-05 |
| Magnoliaceae | **DEC** | **-164.35** | **0.0099** | **0.0001** | **332.72** | **1.00** |
|  | DIVALIKE | -178.33 | 0.012 | 0.0007 | 360.70 | 8.10E-07 |
|  | BAYAREALIKE | -193.94 | 0.0081 | 0.023 | 392.05 | 1.30E-13 |
| Lardizabalaceae | **DEC** | **-40.25** | **0.027** | **0.0043** | **85.11** | **0.99** |
|  | DIVALIKE | -47.24 | 0.037 | 0.0063 | 99.07 | 0.0009 |
|  | BAYAREALIKE | -45.62 | 0.052 | 0.041 | 95.84 | 0.0046 |
| *Celtis* | DEC | -94.66 | 0.036 | 0.027 | 193.64 | 0.013 |
|  | DIVALIKE | -96.52 | 0.03 | 0.0075 | 197.48 | 0.002 |
|  | **BAYAREALIKE** | **-90.31** | **0.049** | **0.099** | **185.04** | **0.99** |
| Elaeagnaceae | DEC | -55.22 | 0.013 | 1.00E-12 | 114.82 | 0.46 |
|  | **DIVALIKE** | **-55.06** | **0.014** | **1.00E-12** | **114.50** | **0.54** |
|  | BAYAREALIKE | -60.89 | 0.015 | 0.034 | 126.24 | 0.0016 |
| Rhamneae | **DEC** | **-324.27** | **0.007** | **0.0008** | **652.63** | **1.00** |
|  | DIVALIKE | -332.55 | 0.0077 | 1.00E-12 | 669.51 | 0.0003 |
|  | BAYAREALIKE | -343.64 | 0.0072 | 0.023 | 691.32 | 3.90E-09 |
| *Cotoneaster* | **DEC** | **-130.66** | **0.072** | **0.013** | **265.42** | **1.00** |
|  | DIVALIKE | -138.45 | 0.09 | 0.02 | 280.12 | 0.0006 |
|  | BAYAREALIKE | -148.47 | 0.06 | 0.1 | 301.45 | 1.90E-08 |
| Ulmaceae | **DEC** | **-69.79** | **0.0034** | **1.00E-12** | **143.90** | **0.89** |
|  | DIVALIKE | -71.98 | 0.0043 | 0.0006 | 148.12 | 0.11 |
|  | BAYAREALIKE | -82.62 | 0.0044 | 0.016 | 169.56 | 2.40E-06 |
| *Acer* | **DEC** | **-212.83** | **0.012** | **0.0043** | **429.76** | **1.00** |
|  | DIVALIKE | -232.44 | 0.012 | 0.0032 | 468.85 | 3.10E-09 |
|  | BAYAREALIKE | -227.35 | 0.015 | 0.025 | 458.74 | 4.90E-07 |
| Altingiaceae-  Hamamelidaceae clade | DEC | -150.13 | 0.0048 | 0.0004 | 304.43 | 1.40E-05 |
|  | DIVALIKE | -157.52 | 0.0047 | 1.00E-12 | 319.12 | 8.50E-09 |
|  | **BAYAREALIKE** | **-138.91** | **0.005** | **0.0096** | **282.02** | **1.00** |

**References**

1. Hai L, Li X, Zhang J *et al*. Assembly dynamics of East Asian subtropical evergreen broadleaved forests: New insights from the dominant Fagaceae trees. *J Integr Plant Biol* 2022; 64: 2126–34.
2. Wang W, Xiang XG, Xiang KL *et al*. A dated phylogeny of Lardizabalaceae reveals an unusual long-distance dispersal across the Pacific Ocean and the rapid rise of East Asian subtropical evergreen broadleaved forests in the late Miocene. *Cladistics* 2020; **36**: 447–57.
3. Li XQ, Peng HW, Xiang KL *et al*. Phylogenetic evidence clarifies the history of the extrusion of Indochina. *Proc Natl Acad Sci USA* 2024; **121**: e2322527121.
4. Yao X, Song Y, Yang JB *et al*. Phylogeny and biogeography of the hollies (Ilex L., Aquifoliaceae). *J Syst Evol* 2021; **59**: 73–82.
5. Jin JJ, Yang MQ, Fritsch PW *et al*. Born migrators: Historical biogeography of the cosmopolitan family Cannabaceae. *J Syt Evol* 2020; **58**: 461–73.
6. Xia XM, Yang MQ, Li CL *et al*. Spatiotemporal evolution of the global species diversity of *Rhododendron*. *Mol Biol Evol* 2022; **39**: msab314.
7. Landis MJ, Eaton DAR, Clement WL *et al*. Joint phylogenetic estimation of geographic movements and biome shifts during the global diversification of *Viburnum*. *Syst Biol* 2021; **70**: 67–85.
8. Katoh K and Standley DM. MAFFT multiple sequence alignment software version 7: Improvements in performance and usability. *Mol Biol Evol* 2013; **30**: 772–80.
9. Kearse M, Moir R, Wilson A *et al*. Geneious Basic: An integrated and extendable desktop software platform for the organization and analysis of sequence data. *Bioinformatics* 2012; **28**: 1647–9.
10. Capella-Gutiérrez S, Silla-Martínez JM, Gabaldón T. trimAl: A tool for automated alignment trimming in large-scale phylogenetic analyses. *Bioinformatics* 2009; **25**: 1972–3.
11. Stamatakis A. RAxML-VI-HPC: Maximum likelihood-based phylogenetic analyses with thousands of taxa and mixed models. *Bioinformatics* 2006; **22**: 2688–90.
12. Wang W, Li HL, Chen ZD. Analysis of plastid and nuclear DNA data in plant phylogenetics—evaluation and improvement. *Sci China Life Sci* 2014; **57**: 280–6.
13. Suchard MA, Lemey P, Baele G *et al*. Bayesian phylogenetic and phylodynamic data integration using BEAST 1.10. *Virus Evol* 2018; **4**: vey016.
14. Rambaut A, Drummond AJ, Xie D *et al*. Posterior summarization in Bayesian phylogenetics using Tracer 1.7. *Syst Biol* 2018; **67**: 901–4.
15. Fu CN, Mo ZQ, Yang JB *et al*. Plastid phylogenomics and biogeographic analysis support a trans-Tethyan origin and rapid early radiation of Cornales in the Mid-Cretaceous. *Mol Phylogenet Evol* 2019; **140**: 106601.
16. Raman G, Choi KS, Lee EM *et al*. Extensive characterization of 28 complete chloroplast genomes of *Hydrangea* species: A perspective view of their organization and phylogenetic and evolutionary relationships. *Comp Struct Biotechnol J* 2023; **21**: 5073–91.
17. Su JX, Dong CC, Niu YT *et al*. Molecular phylogeny and species delimitation of Stachyuraceae: Advocating a herbarium specimen-based phylogenomic approach in resolving species boundaries. *J Syst Evol* 2020; **58**: 710–24.
18. Feng Y, Comes HP, Qiu YX. Phylogenomic insights into the temporal-spatial divergence history, evolution of leaf habit and hybridization in *Stachyurus* (Stachyuraceae). *Mol Phylogenet Evol* 2020; **150**: 106878.
19. Yan Y, Davis CC, Dimitrov D *et al*. Phytogeographic history of the tea family inferred through high-resolution phylogeny and fossils. *Syst Biol* 2021; **70**: 1256–71.
20. Shi XG, Fu QY, Jin JH *et al*. Mummified Oligocene fruits of *Schima* (Theaceae) and their systematic and biogeographic implications. *Sci Rep* 2017; **7**: 4009.
21. Yu XQ, Gao LM, Soltis DE *et al*. Insights into the historical assembly of East Asian subtropical evergreen broadleaved forests revealed by the temporal history of the tea family. *New Phytol* 2017; **215**: 1235–48.
22. Liao M, Shepherd LD, Zhang JY *et al*. Phylogeny, biogeography, and character evolution of the genus *Sophora* s.l. (Fabaceae, Papilionoideae). *Mol Phylogenet Evol* 2023; **181**: 107713.
23. Yan H, Zhou P, Wang W *et al*. Biogeographic history of *Pterocarya* (Juglandaceae) inferred from phylogenomic and fossil data. *J Syst Evol* 2024; **62**: 1165–76.
24. Gandolfo MA, Nixon KC, Crepet WL *et al*. A late Cretaceous fagalean inflorescence preserved in amber from New Jersey. *Am J Bot* 2018; **105**: 1424–35.
25. Manchester SR and Dilcher DL. Reproductive and vegetative morphology of *Polyptera* (Juglandaceae) from the Paleocene of Wyoming and Montana. *Am J Bot* 1997; **84**: 649–63.
26. Belt E, Hartman J, Diemer J *et al*. Unconformities and age relationships, Tongue River and older members of the Fort Union Formation (Paleocene), western Williston Basin, U.S.A. *Rocky Mt Geol* 2004; **39**: 113–40.
27. Manchester SR. The fossil history of the Juglandaceae. *Monogr Syst Bot Mo Bot Gard* 1987; **21**: 1–137.
28. Wang Y, Li E, Sun J *et al*. Phylogenetic diversity and interspecies competition shaped species diversity in adaptive radiated *Ligustrum* (Oleaceae). *J Syst Evol* 2024; **63**: 229–224.
29. Doweld AB. *Palaeoflora Europaea*: Notulae Systematicae ad Palaeofloram Europaeam spectantes I. New names of fossil magnoliophytes of the European Tertiary. I. Miscellaneous families. *Phytotaxa* 2018; **379**: 78–94.
30. Mai DH. Neue Arten nach Früchten und Samen aus dem Tertiär von Nordwestsachsen und der Lausitz. *Feddes Repert* 1987; **98**: 105–26.
31. Mai DH. Die mittelmiozänen und obermiozänen Floren aus der Meuroer und Raunoer Folge in der Lausitz, Teil III: Fundstellen und Paläobiologie. *Palaeontographica Abt B* 2002; **258**: 1–85.
32. Crepet WL, Nixon KC, Gandolfo MA. An extinct calycanthoid taxon, *Jerseyanthus calycanthoides*, from the Late Cretaceous of New Jersey. *Am J Bot* 2005; **92**: 1475–85.
33. Massoni J, Doyle J, Sauquet H. Fossil calibration of Magnoliidae, an ancient lineage of angiosperms. *Palaeontol Electron* 2015; **18.1**: 18.1.2FC.
34. Friis EM, Eklund H, Pedersen KR *et al*. *Virginianthus calycanthoides* gen. et sp. nov.-A Calycanthaceous Flower from the Potomac Group (Early Cretaceous) of Eastern North America. *Int J Plant Sci* 1994; **155**: 772–85.
35. Knight CL and Wilf P. Rare leaf fossils of Monimiaceae and Atherospermataceae (Laurales) from Eocene Patagonian rainforests and their biogeographic significance. *Palaeontologia Electronica* 2013; **16.3**: 16.3.26A.
36. Dillhoff RM, Dillhoff TA, Dunn RE *et al*. Cenozoic paleobotany of the John Day Basin, central Oregon. In: O’Connor JE, Dorsey RJ, Madin IP (eds.). *Volcanoes to Vineyards: Geologic Field Trips through the Dynamic Landscape of the Pacific Northwest*. Geological Society of America, 2009, 135–64.
37. Manchester SR and O’Leary EL. Phylogenetic distribution and identification of fin-winged fruits. *Bot Rev* 2010; **76**: 1–82.
38. Mohr BAR. Palynologische Studien an kretazischen Sedimenten in hohen südlichen Breiten (Legs 113, 114 und 120) und Vorausschau auf Leg 183. In: *ODP-DSDP Kolloquium des DFG-Schwerpunktprogramms, Geologisches Institut, Universität Freiburg, Germany, 1998*. Abstracts, 40.
39. Li HT, Yi TS, Gao LM *et al*. Origin of angiosperms and the puzzle of the Jurassic gap. *Nat Plants* 2019; **5**: 461–70.
40. Ramírez-Barahona S, Sauquet H, Magallón S. The delayed and geographically heterogeneous diversification of flowering plant families. *Nat Ecol Evol* 2020; **4**: 1232–8.
41. Qin SY, Zuo ZY, Guo C *et al*. Phylogenomic insights into the origin and evolutionary history of evergreen broadleaved forests in East Asia under Cenozoic climate change. *Mol Ecol* 2023; **32**: 2850–68.
42. Onstein RE and Linder HP. Beyond climate: Convergence in fast evolving sclerophylls in Cape and Australian Rhamnaceae predates the mediterranean climate. *J Ecol* 2016; **104**: 665–77.
43. Jia DR and Bartish IV. Climatic changes and orogeneses in the Late Miocene of Eurasia: The main triggers of an expansion at a continental scale? *Front Plant Sci* 2018; **9**: 1400.
44. Tian Q, Stull GW, Kellermann J *et al*. Rapid *in situ* diversification rates in Rhamnaceae explain the parallel evolution of high diversity in temperate biomes from global to local scales. *New Phytol* 2024; **241**: 1851–65.
45. Hauenschild F, Favre A, Michalak I *et al*. The influence of the Gondwanan breakup on the biogeographic history of the ziziphoids (Rhamnaceae). *J Biogeogr* 2018; **45**: 2669–77.
46. Gregor HJ. Subtropische Elemente im europäischen Tertiär II (Fruktifikationen). *Paläont Z* 1977; **51**: 199–226.
47. Peppe DJ, Erickson JM, Hickey LJ. Fossil leaf species from the Fox Hills Formation (Upper Cretaceous: North Dakota, USA) and their paleogeographic significance. *J Paleontol* 2007; **81**: 550–67.
48. Calvillo‐Canadell L and Cevallos‐Ferriz SRS. Reproductive structures of Rhamnaceae from the Cerro del Pueblo (Late Cretaceous, Coahuila) and Coatzingo (Oligocene, Puebla) Formations, Mexico. *Am J Bot* 2007; **94**: 1658–69.
49. Meng KK, Chen SF, Xu KW *et al*. Phylogenomic analyses based on genome-skimming data reveal cyto-nuclear discordance in the evolutionary history of *Cotoneaster* (Rosaceae). *Mol Phylogenet Evol* 2021; **158**: 107083.
50. Wolfe JA and Wehr W. *Middle Eocene dicotyledonous plants from Republic, northeastern Washington*. Reston, VA: Department of the Interior, U.S. Geological Survey; 1987.
51. Zhang QY, Deng M, Bouchenak-Khelladi Y *et al*. The diversification of the northern temperate woody flora – A case study of the Elm family (Ulmaceae) based on phylogenomic and paleobotanical evidence. *J Syt Evol* 2022; **60**: 728–46.
52. Denk T and Grimm GW. Phylogeny and biogeography of *Zelkova* (Ulmaceae *sensu stricto*) as inferred from leaf morphology, ITS sequence data and the fossil record. *Bot J Linn Soc* 2005; **147**: 129–57.
53. Feng GP, Ablaev AG, Wang YF *et al*. Paleocene Wuyun Flora in Northeast China: *Ulmus furcinervis* of Ulmaceae. *Acta Bot Sin* 2003; **45**: 146–51.
54. MacGinitie HD. *A Middle Eocene flora from the Central Sierra Nevada*. Washington, DC: Carnegie Institution of Washington; 1941.
55. Wang Q, Manchester SR, Li C *et al*. Fruits and Leaves of *Ulmus* from the Paleogene of Fushun, Northeastern China. *Int J Plant Sci* 2010; **171**: 221–6.
56. Myers HW and Manchester SR. *The Oligocene Bridge Creek Flora of the John Day Formation, Oregon*. Berkeley: University of California Publications in Geological Sciences; 1997.
57. MacGinitie HD. *Fossil plants of the Florissant beds, Colorado*. Washington, DC: Carnegie Institution of Washington; 1953.
58. Manchester SR. Systematics and fossil history of the Ulmaceae. In: Crane PR and Blackmore S (ed.). *Evolution, Systematics, and Fossil History of the Hamamelidae*. Oxford: Clarendon Press, 1989, 221–51.
59. Renner SS, Grimm GW, Schneeweiss GM *et al*. Rooting and dating maples (*Acer*) with an uncorrelated-rates molecular clock: Implications for North American/Asian disjunctions. *Syst Biol* 2008; **57**: 795–808.
60. Li J, Stukel M, Bussies P *et al*. Maple phylogeny and biogeography inferred from phylogenomic data. *J Syst Evol* 2019; **57**: 594–606.
61. McClain AM and Manchester SR. *Dipteronia* (Sapindaceae) from the Tertiary of North America and implications for the phytogeographic history of the Aceroideae. *Am J Bot* 2001; **88**: 1316–25.
62. Wolfe JA and Tanai T. Systematics, phylogeny, and distribution of *Acer* (maples) in the Cenozoic of Western North America. *J Fac Sci Hokkaido Univ* 1987; **22**: 1–246.
63. McClain AM. Systematics and biogeography of selected modern and fossil *Dipteronia* and *Acer* (Sapindaceae). *Master Thesis*. University of Florida, 2000.
64. Areces-Berazain F, Hinsinger DD, Strijk JS. Genome-wide supermatrix analyses of maples (*Acer*, Sapindaceae) reveal recurring inter-continental migration, mass extinction, and rapid lineage divergence. *Genomics* 2021; **113**: 681–92.
65. Xiang X, Xiang K, Ortiz RDC *et al*. Integrating palaeontological and molecular data uncovers multiple ancient and recent dispersals in the pantropical Hamamelidaceae. *J Biogeogr* 2019; **46**: 2622–31.
66. Mai DH. The fossils of *Rhodoleia* Champion (Hamamelidaceae) in Europe. *Acta Palaeobot* 2001; **41**: 161–75.
67. Collinson ME, Manchester SR, Wilde V. Fossil fruits and seeds of the Middle Eocene Messel biota, Germany. *Abh Senckenberg Ges Naturforsch* 2012; **570**: 1–249.
68. Tanai T. On the Hamamelidaceae from the Palaeogene of Hokkaido, Japan. *Trans Proc Palaeont Soc Japan* 1967; **1967**: 56–62.
69. Magallon-Puebla S, Herendeen PS, Endress PK. *Allonia decandra*: Floral remains of the tribe Hamamelideae (Hamamelidaceae) from Campanian strata of southeastern USA. *Plant Syst Evol* 1996; **202**: 177–98.
70. Magallón S. From fossils to molecules: Phylogeny and the core eudicot floral froundplan in Hamamelidoideae (Hamamelidaceae, Saxifragales). *Syst Bot* 2007; **32**: 317–47.
71. Li J. Molecular phylogenetics of Hamamelidaceae: Evidence from DNA sequences of nuclear and chloroplast genomes. In: Sharma AK and Sharma A (eds.). *Plant Genome Biodiversity and Evolution*. Delhi: Science Publishers, 2008, 227–50.
72. Radtke MG, Pigg KB, Wehr WC. Fossil *Corylopsis* and *Fothergilla* leaves (Hamamelidaceae) from the Lower Eocene flora of Republic, Washington, U.S.A., and their evolutionary and biogeographic significance. *Int J Plant Sci* 2005; **166**: 347–56.
73. Ozaki K. Two new fossil species of *Fortunearia* and *Davidia* from the upper Motojuku (Kabutoiwa) Formation in central Japan. *Mem Inst Field Educ, Yokohama Natl Univ* 1984; **2**: 1–8.
74. Endress PK and Friis EM. *Archamamelis*, hamamelidalean flowers from the Upper Cretaceous of Sweden. *Plant Syst Evol* 1991; **175**: 101–14.
75. Maslova NP. Genus *Parrotiopsis* (Niedz.) Schneid. (Hamamelidaceae R. Brown)—the first record in the Tertiary of Eastern Asia. *Paleontol J* 1995; **29**: 159–66.
76. Hu HH and Chaney RW. A Miocene flora From Shantung Province, China. Washington, DC: Carnegie Institution of Washington, 1940.
77. Wang XQ and Li HM. Discovery of another living fossil – *Shaniodendron subaequale* (H.T. Chang) Deng *et al*. in China clearing up paleobotanists’s a long-term doubt. *Acta Palaeontol Sin* 2000; **39**: 308–17.
78. Ree RH and Sanmartín I. Conceptual and statistical problems with the DEC+J model of founder-event speciation and its comparison with DEC via model selection. *J Biogeogr* 2018; **45**: 741–9.
79. Matzke NJ. Statistical comparison of DEC and DEC+J is identical to comparison of two ClaSSE submodels, and is therefore valid. *J Biogeogr* 2022; **49**: 1805–24.
80. Matzke NJ. Probabilistic historical biogeography: New models for founder-event speciation, imperfect detection, and fossils allow improved accuracy and model-testing. *Front Biogeogr* 2013; **5**: 242–8.
81. Yu Y, Blair C, He X. RASP 4: Ancestral state reconstruction tool for multiple genes and characters. *Mol Biol Evol* 2020; **37**: 604–6.
82. Szekely G and Rizzo M. Hierarchical clustering via joint between-within distances: Extending Ward’s minimum variance method. *J Classif* 2005; **22**: 151–83.
83. James NA and Matteson DS. ecp: An R package for nonparametric multiple change point analysis of multivariate data. *J Stat Softw* 2015; **62**: 1–25.
84. Song YC and Da LJ. Evergreen Broad-Leaved Forest of East Asia. In: Box EO (ed.). *Vegetation Structure and Function at Multiple Spatial, Temporal and Conceptual Scales*. Cham: Springer, 2016, 101–28.
85. Wickham H. ggplot2: Elegant Graphics for Data Analysis. New York, NY: Springer-Verlag; 2016.
86. Hagen O, Skeels A, Onstein RE *et al*. Earth history events shaped the evolution of uneven biodiversity across tropical moist forests. *Proc Natl Acad Sci USA* 2021; **118**: e2026347118.
87. Pinheiro J and Bates D. *nlme: Linear and nonlinear mixed effects models*. https://cran.r-project.org/web/packages/nlme/index.html (30 April 2026, data last accessed).
88. Farnsworth A, Lunt DJ, Robinson SA *et al*. Past East Asian monsoon evolution controlled by paleogeography, not CO_2_. *Sci Adv* 2019; **5**: eaax1697.
89. Westerhold T, Marwan N, Drury AJ *et al.* An astronomically dated record of Earth’s climate and its predictability over the last 66 million years. *Science* 2020; **369**: 1383–7.
90. Fox J and Weisberg S. An R Companion to Applied Regression. Thousand Oaks, CA: SAGE Publications; 2019.
91. Lindeløv JK. mcp: An R package for regression with multiple change points. *OSF Preprints* 2020, doi: 10.31219/osf.io/fzqxv.
92. Wootton LM, Boucher FC, Pouchon C *et al*. The late rise of sky-island vegetation in the European Alps. *Nat Plants* 2025; **11**: 1142–53.
93. Vehtari A, Gelman A, Gabry J. Practical Bayesian model evaluation using leave-one-out cross-validation and WAIC. *Stat Comput* 2017; **27**: 1413–32.
94. APG IV. An update of the Angiosperm Phylogeny Group classification for the orders and families of flowering plants: APG IV. *Bot J Linn Soc* 2016; **181**: 1–20.
